# Signaling pathways and targeted therapies for psoriasis

**DOI:** 10.1038/s41392-023-01655-6

**Published:** 2023-11-27

**Authors:** Jia Guo, Hanyi Zhang, Wenrui Lin, Lixia Lu, Juan Su, Xiang Chen

**Affiliations:** 1 Department of Dermatology, Xiangya Hospital, Central South University, No.87 Xiangya Road, Changsha, 410008 Hunan China; 2 National Engineering Research Center of Personalized Diagnostic and Therapeutic Technology, Changsha, 410008 Hunan China; 3 Hunan Key Laboratory of Skin Cancer and Psoriasis, Changsha, 410008 Hunan China; 4 Hunan Engineering Research Center of Skin Health and Disease, Changsha, 410008 Hunan China

**Keywords:** Inflammation, Immunopathogenesis, Therapeutics

## Abstract

Psoriasis is a common, chronic, and inflammatory skin disease with a high burden on individuals, health systems, and society worldwide. With the immunological pathologies and pathogenesis of psoriasis becoming gradually revealed, the therapeutic approaches for this disease have gained revolutionary progress. Nevertheless, the mechanisms of less common forms of psoriasis remain elusive. Furthermore, severe adverse effects and the recurrence of disease upon treatment cessation should be noted and addressed during the treatment, which, however, has been rarely explored with the integration of preliminary findings. Therefore, it is crucial to have a comprehensive understanding of the mechanisms behind psoriasis pathogenesis, which might offer new insights for research and lead to more substantive progress in therapeutic approaches and expand clinical options for psoriasis treatment. In this review, we looked to briefly introduce the epidemiology, clinical subtypes, pathophysiology, and comorbidities of psoriasis and systematically discuss the signaling pathways involving extracellular cytokines and intracellular transmission, as well as the cross-talk between them. In the discussion, we also paid more attention to the potential metabolic and epigenetic mechanisms of psoriasis and the molecular mechanistic cascades related to its comorbidities. This review also outlined current treatment for psoriasis, especially targeted therapies and novel therapeutic strategies, as well as the potential mechanism of disease recurrence.

## Introduction

Psoriasis is a common, chronic, and inflammatory skin disease affecting approximately 2–3% of the global population. It has been reported that the incidence of psoriasis varies from 30.3 per 100,000 person-years to 321.0 per 100,000 person-years and is associated with age, sex, geographical location, ethnicity, and other genetic and environmental factors.^1–3^ Psoriasis can occur at any age, especially in two age group of 16–22 and 55–60 years. It also can significantly impact the quality of life and sources of income for the patients.^2,4^ An epidemiological study suggested that severe psoriasis could increase the loss of work productivity by more than four times, which is associated with a greater impact on quality of life (DLQI > 10), younger age (<40 years), and joint involvement.^4^ The severe economic burden has been found in multiple countries around the world and is correlated with the severity of the disease.^5–8^ Patients with psoriasis are also found to be significantly more prone to suicidal ideation (OR = 2.05, 95% CI: 1.54–2.74), suicide attempt (OR = 1.32, 95% CI: 1.14–1.54), and completed suicide (OR = 1.20, 95% CI: 1.04–1.39).^9,10^ In brief, psoriasis is a prevalent and costly disease that imposes a significant burden on individuals, health systems, and society worldwide.

**Fig. 1:**
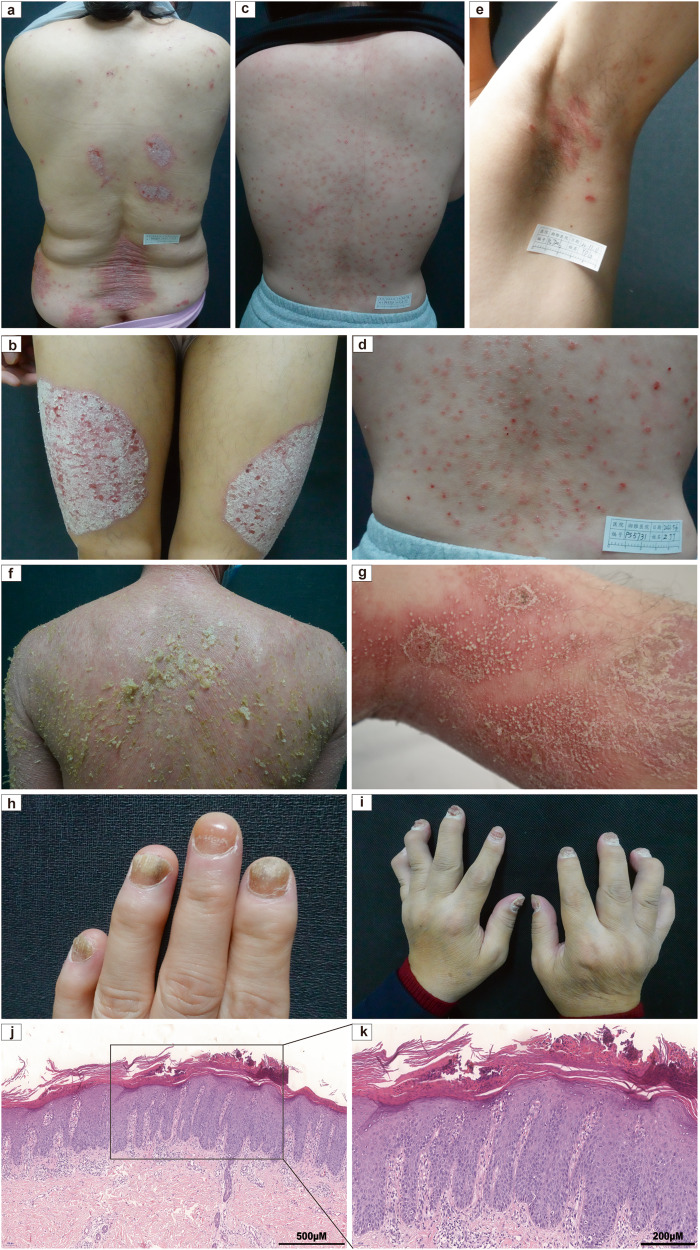
Clinical diversity and histopathological features of psoriasis. **a**, **b** Plaque psoriasis, **c**, **d** Guttate psoriasis, **e** inverse psoriasis, **f** erythrodermic psoriasis, **g** pustular psoriasis, **h** Nail pitting, **i** psoriatic arthritis, 100× (**j**) and 200× (**k**) of histopathological images of psoriasis

The etiology of psoriasis is complex and still remains largely unclear; it involves genetic susceptibility, environmental triggers, and immune dysregulation.^11–13^ Psoriasis is a heterogeneous disease that can be classified into different clinical types, with the most common type being plaque psoriasis (also known as psoriasis vulgaris), which causes dry, itchy, and raised skin patches (i.e., plaques) with silvery scales.^14,15^ These plaques can occur at any site of the skin but are more common on the elbows, knees, scalp, and lower back. Other types of psoriasis include guttate psoriasis, inverse psoriasis, pustular psoriasis, and erythrodermic psoriasis (Fig. 1).^12,16–19^ A growing body of research has indicated that psoriasis is a systemic disease with a variety of comorbidities, such as cardiovascular diseases, metabolic syndrome, psoriatic arthritis, depression, and anxiety.^11,20–22^ Therefore, attention needs to be given to comorbidities of psoriasis as well as the disease per se to work out early intervention and treatment strategies.

To date, the diagnosis of psoriasis is mainly based on the clinical features of skin lesions, such as morphology, location, and scaling status. A skin biopsy can be performed to confirm the diagnosis or exclude other conditions.^23–25^ The most common microscopic features of psoriasis vulgaris are hyperkeratosis with parakeratosis and neutrophil microabscesses in the parakeratosis region (also known as Munro’s microabscesses). It is also characterized by significantly reduced or absent granular layer, thinning of the muscular layer above the dermal papilla, extending or shifting telangiectasia, and infiltration of lymphocytes and neutrophils around the lesions.^12,26,27^ Psoriatic arthritis can be diagnosed by a rheumatologist through clinical examination, medical imaging, and laboratory tests.^28,29^ The treatment of psoriasis depends on the severity, extent, and type of psoriasis, as well as the patient’s preferences and comorbidities. The primary goals of treating psoriasis are to reduce inflammation, clear skin lesions, improve quality of life, and prevent complications. Conventional therapies for psoriasis include corticosteroids, vitamin D analogs, calcineurin inhibitors, phototherapy, methotrexate, cyclosporine, acitretin, and apremilast.^11,24,28^ During the past decade, injectable biologics targeting specific molecules involved in the pathogenesis of psoriasis have gradually been developed or studied. They include TNF-alpha inhibitors (adalimumab, etanercept, infliximab), IL-12/23 inhibitors (ustekinumab), IL-17 inhibitors (secukinumab, ixekizumab), and IL-23 inhibitors (brodalumab), and so on.^30,31^

In this review, we looked to summarize and discuss the signaling pathways involved in psoriasis and their roles, the clinical diversity of psoriasis and associated comorbidities, pathophysiology, epidemiology, targeted therapies, as well as clinical research progress. We hope to provide a reference for future studies on and personalized treatment of psoriasis.

## Signaling pathways driving psoriasis pathogenesis

The pathogenesis of psoriasis has gradually been elucidated in the past years. Studies have revealed that psoriasis is regulated by the complex interactions between extracellular cytokine pathways and intracellular signaling molecules.^32^ A variety of cytokines transmit extracellular signals to the cell membrane and then are recognized by related receptors, leading to the activation of intracellular signaling pathways and inducing a series of events that ultimately result in the inflammatory signaling cascade.^33,34^ Epigenetics also plays a critical role in the pathogenesis of the psoriasis.^35,36^ Furthermore, recent studies also suggested that metabolic reprogramming is emerging as another crucial regulatory paradigm for psoriasis.^37–39^

### Extracellular cytokine pathways

#### The TNF/IL-23/IL-17 pathway

The findings of a large number of genome-wide association studies (GWAS) and clinical trials support the central role of TNF-IL-23-IL-17 signaling pathways in the pathogenesis of psoriasis, especially for plaque psoriasis.^40–47^

#### IL-23

Interleukin-23 (IL-23) is a heterodimeric protein constituted by the p19 and the p40 subunits via a disapplied bond.^48^ Prior studies have revealed that IL-12 and IL-23 share the p40 subunit; however, only IL-23 uses the p19 subunit, while IL-12 uses the p35 subunit. Both IL-23 and IL-12 belong to the IL-12 family.^49^ IL-12 and IL-23 are mainly secreted by dendritic cells (DCs), the levels of which are elevated in psoriasis. A growing body of evidence has suggested that the RNA expression of both p40 and p19 increases greatly in psoriatic lesions, while the expression of p35 mRNA remains largely unchanged.^50,51^ The level of IL-23 protein can be greatly elevated in psoriatic lesions compared to unaffected skin.^50^ Thus, it can be inferred that the pathogenesis of psoriasis is related to IL-23 rather than IL-12.

IL-23 acts on T cells, especially the CD4^+^ helper T cells (Th17 cells), via a cellular receptor complex comprising of two transmembrane proteins: IL-23Rα and IL-12Rβ1.^52^ Then, IL-23 promotes the release of interleukin-17 (IL-17), another critical cytokine involved in the pathogenesis of psoriasis, by Th17 cells through the activation of intracellular signals.^53^

#### IL-17

The IL-17 family includes six structurally related cytokines: IL-17A to IL-17F.^54^ Among them, it is reported that IL-17A, IL-17C, and IL-17F are related to the pathogenesis of psoriasis due to their increased expression in psoriatic lesions.^55–57^ Although IL-17C and IL-17F exhibited higher levels of expression in psoriasis, IL-17A is found to be the most biologically active cytokine in this family.^55,58^ IL-17A and IL-17F can function as homodimers or IL-17A/F heterodimers.^59,60^ To date, IL-17A (commonly known as IL-17) has received the greatest attention due to its pro-inflammatory role in autoimmune disease.^61^ Although it is mainly produced by Th17 cells. Recently, innate immune cells, including ILC3 cells, γδT cells, neutrophils, and mast cells, have also been found to source IL-17 in psoriasis.^62^

Similar to IL-23, IL-17 also acts on its target cells through corresponding receptor complexes. The signals of IL-17A/IL-17A and IL-17F/IL-17F homodimers and IL-17A/IL-17F heterodimers transmit via IL-17RA and IL-17RC transmembrane complex. The IL-17RA chain is also the co-receptor of IL-17C (when binding to IL-17RE) and IL-17E (when binding to IL-17RB).^63^ A recent study has revealed that CMTM4 is a new component of the IL-17RC. It can mediate the plasma membrane localization of IL-17RC and the subsequent transduction of IL-17A and IL-17F signaling.^64^ The released IL-17, especially IL-17A and IL-17F, mainly acts directly on keratinocytes to stimulate the production of some molecules such as cytokines, antimicrobial peptides (AMPs), and β-defensins, as well as chemokines, including chemokine (C-X-C motif) ligand 1 (CXCL1), CXCL2, CXCL8, and CCL20; these molecules are often increased in psoriatic lesions to attract neutrophils, macrophages and lymphocytes.^65^ There is also research finds IL-17A can upregulate Galectin-8, which further promotes keratinocytes proliferation by regulating its mitosis.^66^ S100A8 and S100A9, as reliable biomarkers of psoriasis disease activity, are highly up-regulated in keratinocytes. It has been investigated that IL-17A and IL-17F can highly induce S100A8 and S100A9 expression and release.^67^ Moreover, IL-17 can promote keratinocyte stemness and then promote keratinocyte proliferation.^68^ Except for acting on keratinocytes, recent studies have revealed that IL-17A can also be involved in psoriasis via regulating other stromal cells, T cells, or monocytes. On the one way, IL-17A directly induces IL-19 and IL-24 expression of fibroblast cells in the skin via binding with IL-17RA and IL-17RC, which further contributes to keratinocyte proliferation and acanthosis.^69^ On the other way, the released IL-17A also acts on other immune cells to facilitate psoriasis pathogenesis. Liu et al. have found that IL-17A can block the suppressive function of Tregs.^70^

**Fig. 2:**
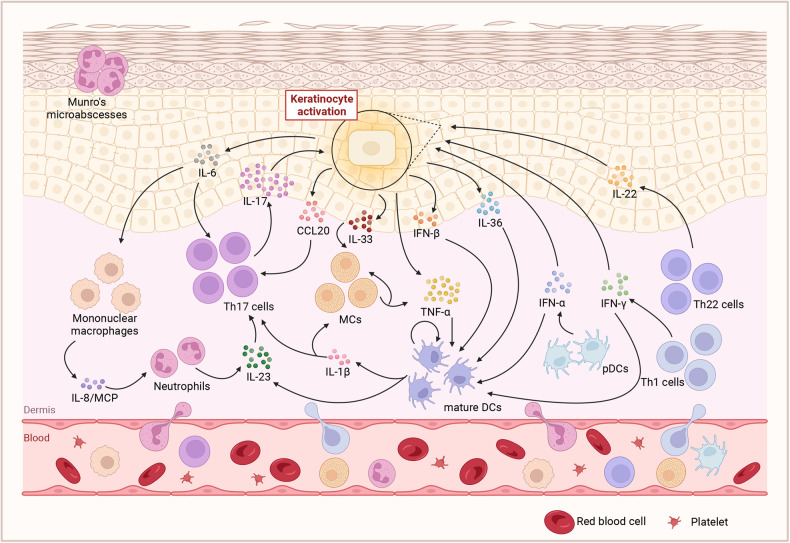
The cross-talk of extracellular cytokine pathway in psoriasis. A complex network links the essential cells and molecules in the pathogenesis of psoriasis. And the cross-talk is considered the core of the progress. On the one hand, the mutual promotion of adaptive and innate immune system produces several cytokines and maintain psoriatic hallmark features in both the dermis and epidermis. On the other hand, the keratinocytes facilitate the inflammatory mediators and enhance the expansion of local activation. Created with BioRender.com. Abbreviations: MCs mast cells, pDCs plasmacytoid dendritic cells, mature DCs mature dendritic cells, MCP Monocyte chemotactic protein, CCL20 Chemokine (C-C motif) ligand 20

Unlike IL-17A and IL-17F, IL-17C is primarily released by keratinocytes. Previous studies have suggested that IL-17C is produced by keratinocytes after stimulation by TNF-α or toll-like receptor agonists and then exerts the effects of IL-17A and IL-17F.^71^ IL-17C can also stimulate the production of human β-defensin 2 and granulocyte colony-stimulating factor (Fig. 2).^72^ As for IL-17E, it acts as a crucial factor in recruiting innate immune cells like monocytes/macrophages and neutrophils to the skin. What’s more, it targets keratinocytes in an autocrine manner and then induces keratinocyte proliferation via the mTOR signaling pathway in psoriasis.^73^

#### TNF-α

Tumor necrosis factor (TNF)-α is another critical inflammatory cytokine that is highly expressed in psoriatic lesions. This cytokine plays a pivotal role in the pathogenesis of psoriasis, which is also demonstrated by the efficacy of TNF-α-targeted therapies.^74,75^ TNF-α is produced by a variety of cells related to the development of psoriasis, such as keratinocytes, DCs, neutrophils, mast cells, as well as NKT, Th1, Th17 and Th22 cells.^76,77^ It acts on the targeted cells mainly via two types of TNF receptors, i.e., TNFRI (p55) and TNF-RII (p75). It has been reported that TNF-α exerts its biological effect on epidermal cells by binding to the p55 TNF-R.^78^On the one hand, TNF-α significantly suppresses the secretion of IFN-α of plasmacytoid dendritic cells (pDCs).^79^ It is the main reason for the occurrence of aggravated psoriasis or new paradoxical psoriasis during treatment with TNF-α inhibitors.^80^ On the other hand, TNF-α favors pDCs maturation to a more conventional dendritic cell phenotype to produce IL-23^81^; Moreover, TNF-α can induce the synthesis of IL-12 and IL-18, two cytokines that are potent inducers of IFN-γ, to participate in the regulation of the Th1 immune response.^82^ Furthermore, TNF-α acts synergistically with IL-17A to coregulate psoriasis-related cytokines and keratinocyte genes, affecting the function of keratinocytes.^83^ All of the above findings suggest that TNF-α is an important regulator of the IL-23/IL-17 axis. Recently, research reveals that TWEAK displayed a similar effect with TNF-α and IL-17A on up-regulating the expression of CXC chemokines, along with cytokines such as IL-23 and inflammation-associated proteins like S100A8/9. Moreover, it may act with TNF and IL-17 to enhance the feedback inflammatory activity of keratinocytes. Hence, TWEAK inhibition may be comparable to targeting TNF or IL-17. Blocking TWEAK may be considered an alternate therapy for psoriasis.^84^

As can be seen, on the one way, the released TNF-α facilitates the release of IL-23 by DCs. On the other way, TNF-α also interacts with keratinocytes to release inflammatory cytokines and promote T-cell infiltration. Then, the secreted IL-23 acts on T cells, especially Th17 cells, to promote the release of IL-17 by binding to the IL-23 receptors. Furthermore, IL-17 acts synergistically with TNF-α to stimulate keratinocytes to produce inflammatory cytokines and chemokines to recruit more inflammatory cells, thus amplifying the inflammatory cascade, leading to the development of psoriasis. The central role of the TNF-α/IL-23/IL-17 axis in the pathogenesis of psoriasis has been demonstrated in numerous studies (Fig. 2).

#### The CCL20-CCR6 pathway

CCL20, also known as MIP-3α or LARC, is an important chemokine in psoriasis. Unlike other chemokines that have multiple receptors, CCL20 binds to only one receptor, CCR6.^85^ It is observed that CCL20 is increased in the serum and lesions of patients with psoriasis,^86^ which has been further confirmed in an in vivo study showing the upregulation of CCL20/CCR6 in psoriasis mouse models. It is found that the level of CCL20 and CCR6^+^T cells increases greatly in the imiquimod-induced psoriasis mouse model and that CCR6^+^ γδ T cells infiltrate in the skin producing IL-17A and IL-22 in the IL-23-induced psoriasis model.^87,88^ Thus, it seems that the CCL20/CCR6 axis plays a vital role in the pathogenesis of psoriasis.

The major source of CCL20 is keratinocytes, while CCR6 is mainly expressed in DCs and T cells, especially Th17 cells.^89^ A recent study suggested that CCR6 is a representative maker for Th17 cells.^90^ It is found that keratinocyte transglutaminase 2 (TG2) can upregulate CCL20 expression, promoting the recruitment of CCR6^+^ γδ T cells.^91^ Moreover, CCL20 can be upregulated by several cytokines, such as IL-17A and TNF-α, in keratinocytes.^65,92^ Cellular dissociation by scratching or trypsinization is also found to be a potent stimulator for the rapid production of CCL20 in keratinocytes.^93,94^

In brief, external factors such as scratching or cytokines can stimulate the production of CCL20 by keratinocytes in psoriatic lesions. Subsequently, the released CCL20 recruits CCR6^+^ Th17 cells, which produce IL-17A, further enhancing the selection of CCL20. Thus, the CCL20/CCR6 axis works as a driving force in the maintenance of the TNF-α/IL-23/IL-17A axis (Fig. 2).

#### The IL-36/IL-1 pathway

In addition to the TNF-α-IL-23-IL-17 axis, the IL-36-IL-1 inflammatory axis is also predominant in the pathogenesis of psoriasis, especially the generalized pustular psoriasis (GPP).^95^

IL-36 comprises of three agonists, namely IL-36α, IL-36β, and IL-36γ, and two antagonists, i.e., the IL-36 receptor antagonist (IL-36RN) and IL-38.^96^ IL-36 belongs to a subgroup in the wider IL-1 family,^97^ which also include IL-1α, IL-1β, IL-1 receptor antagonist (IL-1RN), IL-18, IL-33, and IL-37.^98^ Johnston et al. have reported that the expression of IL-36α, IL36β, IL-36γ, and IL-36Ra is up-regulated in psoriasis and can further promote the expression of keratinocyte antimicrobial peptide, indicating their importance in psoriasis.^99^ Moreover, the identification of mutations in IL-36 signature genes, especially the IL-36RN gene, confirmed the pathogenic role of the IL-36 axis in the development of psoriasis.^100–102^ In 2015, IL-1RL1 was identified as a new susceptibility locus for psoriasis.^103^ Further genetic studies indicated that polymorphisms of the IL-1B gene could be used to distinguish between early- and late-onset psoriasis.^104^

Keratinocytes have been identified as the predominant source of IL-36, which acts on target cells through the receptor, IL-36R (also known as IL-1RL2 or IL-1Rrp2).^105^ It has been demonstrated that the IL-36 cytokines need the N-terminal peptide cleavage to activate.^106^ The activation can be carried out by the serine proteases, including cathepsin G, elastase, and proteinase 3, which are derived from neutrophils. Besides, it can also be activated by cathepsin S released from keratinocytes and fibroblasts.^107,108^ During this progress, the activation of IL-36 can be inhibited by protease inhibitors, including α1-antitrypsin and α1-antichymotrypsin. More interestingly, α1-antitrypsin and α1-antichymotrypsin are encoded by SERPINA1 and SERPINA3, and the latter is one of the most common mutations molecular in pustular psoriasis.^95^ Further studies have shown that activating IL-36 promotes the production of neutrophil-attracting chemokines (CXCL1, CXCL2, CXCL8), other T cell chemokines, macrophage chemokines, and antimicrobial peptides; this process seems to be a self-amplifying loop.^109,110^ Moreover, mature IL-36 induces T-cell proliferation and Th-1 and Th-17 cell differentiation.^111,112^ In addition, the activated IL-36 also acts on dendritic cells to support their phenotypic and functional maturation and trigger the production of IL-1β, TNF-α, and IL-23.^32,113^ Recent research has investigated that a novel soluble IL-36R (sIL-36R), which is encoded similar ectodomain of IL-36R, can suppress psoriasis inflammation by binding IL-36γ and competing with IL-36R for IL-36γ binding in a dose-dependent manner. The production of sIL-36R is mainly regulated by the IL-36 pre-mRNA splicing mediated by DDX5. Moreover, the activated IL-17D signaling inhibits the expression of DDX5. This study provides new insight into how a novel soluble IL-36R participates in psoriasis inflammation.^114^

IL-1β is the main form of IL-1 in circulation. Like other cytokines in the IL-1 family, it has an inactive precursor that requires the caspase-1 cleavage or neutrophil-derived serine proteases to be biologically activated.^115^ DCs, monocytes, and macrophages are the main sources of the expression, activation, and secretion of IL-1β. It is reported that the level of IL-1β expression is elevated in psoriatic lesions and correlated with the severity of disease and treatment response. IL-1β has been demonstrated to play a critical role in the differentiation of Th17 and γδT17 cells. It can also stimulate keratinocytes to secrete chemokines such as CCL20, which is an important chemokine increase in psoriasis (Fig. 2).^116^

#### The IFN pathway

There are three subtypes of IFNs, i.e., type I (IFN-α and IFN-β), type II (IFN-γ), and type III (IFN-λ).^117^ IFNs of type I and type III can be released by several types of cells, such as epithelial cells, macrophages, and DCs, while the type II IFN is secreted by activated NK and T cells only.^118,119^ Recently, it has been highly recognized that IFNs serve as vital mediators in the pathogenesis of psoriasis, with type I and type II IFNs being the main mediators. Type I IFNs are key cytokines related to chronic viral infection, which often triggers psoriasis.^120^ Thus, treatment with type I IFNs often induces or exacerbates psoriasis or psoriatic arthritis.^121,122^ Preclinical experiments also found that mice lacking type 1 IFN receptor or treated with IFN-α or IFN-β antibodies failed the treatment for Th17-mediated psoriasis-like inflammation.^123^ Previously, psoriasis was regarded as an IFN-γ-mediated disease due to the significant IFN-γ genomic signature in psoriatic lesions.^124^ The main IFN-γ-producing cells, Th1 cells, are also considered an integral part of the pathogenesis of psoriasis.^125^ Prior studies have found a positive correlation between serum IFN-γ levels and the severity of disease assessed using the Psoriasis Area and Severity Index (PASI). Hence, IFN-γ seems to be a prognostic factor of this disease.^126^ All of the above findings indicate that the IFN pathway plays a critical role in the pathogenesis of psoriasis.

Studies have shown that the innate immune cells such as skin-resident keratinocytes and infiltrated plasmacytoid dendritic cells (pDCs) play a significant role in initiating subsequent adaptive immune events, including DCs maturation and T cell activation.^127,128^ Gilliet et al. found that pDCs are the natural IFN-α producing cells; pDCs infiltrate the skin and are activated to produce IFN-α at the early stage of psoriasis.^129^ With regard to IFN-β, numerous studies have recognized keratinocytes as the primary source of this cytokine.^130^ Along with IFN-α, IFN-β can also trigger the maturation and differentiation of conventional dendritic cells (cDCs) via IFNAR1 and IFNAR2 receptors on the target cells.^131^ Subsequently, the mature DCs release cytokines such as TNF-α, IL-23, and IL-1β to activate Th17 cells. Moreover, IFN-α can increase the expression of IL-22 receptors on keratinocytes, enhancing the response of keratinocytes to IL-22.^132^

The main source of IFN-γ is activated Th-1 cells. Besides, IFN-γ can also be secreted by DCs, CD8 T cells, and NK cells.^133^ The secreted IFN-γ activates their effect cells by binding IFN-γ receptors (IFNGRs), which are composed of two transmembrane chains: IFN-γ R1 and IFN-γ R2.^133^ The production of IFN-γ can be regulated by IL-12, IL-18, and IL-23. Moreover, IL-23 has a synergistic effect with IL-18 for IFN-γ production.^134,135^ In psoriasis, IFN-γ acts on keratinocytes, leading to the activation and production of antimicrobial peptides such as LL-37 cathelicidin and β-defensins.^125^ Recent studies also found that IFN-γ could induce the release of cytokines (IL-23 and IL-1) and adhesion molecules of DCs, which activate the Th17 cells.^136^

In summary, the IFN pathway plays an important role in the inflammatory cascade in the pathogenesis of psoriasis (Fig. 2).

### Other cytokines and pathways related to psoriasis

#### IL-22

IL-22 belongs to the IL-10 family, which also includes IL-10, IL-19, IL-20, IL-24 and IL-26.^137^ Prior studies have found that the level of IL-22 is significantly increased in the serum and lesions of patients with psoriasis.^138,139^ The concentration of serum IL-22 is also significantly correlated with the PASI score and is regarded as an indicator of therapeutic effect, as treatment with etanercept or methotrexate can reduce the level of serum IL-22. This also indicates that serum IL-22 is a potential predictor for the severity of psoriasis.^140,141^

IL-22 can be secreted by a variety of immune cells, such as Th17 cell, Th1 cell, Th22 cell, γδT cell, and NK cells.^142–144^ The secreted IL-22 acts on its target cells by binding to IL-22 receptors, which consist of two subunits, IL-22R1 and IL-10R2 (also known as IL10RB).^145^ It has been demonstrated that IL-22 does not act on immune cells because IL-22R1 is only expressed on non-immune cells such as keratinocytes.^146^ The binding of IL-22 to IL-22R leads to the activation of downstream signals (which will be presented in the next section) in keratinocytes, inducing the production of antimicrobial proteins.^146^ Furthermore, IL-22 can inhibit the differentiation of keratinocytes, leading to acanthosis, a typical psoriasis-like inflammation of epidermis.^147^ Interestingly, TNF-α can enhance this effect of IL-22.^147^ However, the therapeutic inhibition of IL-22 might be insufficient for a good clinical response according to early clinical trials on anti-IL-22 monoclonal antibodies (ILV-095 and ILV-095) in patients with psoriasis, which were discontinued for their failure to meet the primary efficacy endpoint (ClinicalTrials.gov identifier: NCT00563524 and NCT01010542).

#### IL-6

IL-6, a traditional cytokine in psoriasis, may exert a controversial effect on psoriasis. During the past two decades, IL-6 has been reported to be associated with the pathogenesis of psoriasis.^133,148^ But only till recently, the expression of the IL-6 gene has been regarded as a marker for the pathological state of psoriasis and psoriatic arthritis.^149^ In psoriasis, the source of IL-6 includes almost all stromal cells, including keratinocytes, fibroblasts, endothelial cells, and immune cells such as dendritic cells, macrophages, and Th17 cells.^150,151^ IL-6 acts through interacting with IL-6R. The fully competent IL-6R consists of an alpha subunit IL-6R (CD126) and a commonly expressed beta subunit gp130 (CD130).^152,153^ Recent evidence demonstrates that IL-6 can initiate the development of Th17 cells.^154^ The IL-17 generated by Th17 cells can feedback regulate the production of IL-6 of keratinocytes.^83^ The positive feedback loop of IL-6/IL-17 signaling participates in the proinflammatory interaction of innate and adaptive immunity in patients with psoriasis. Moreover, IL-6 can promote neutrophil migration by increasing the level of chemokines released by mononuclear cells/macrophages, such as IL-8 and MCP-1.^153^ Mature neutrophils also release pro-inflammatory cytokines such as IL-23 and IL-17 by responding to IL-6 through the membrane-bound IL-6R, which helps to establish a positive feedback loop for Th17 polarization.^148^ Based on these findings, IL-6 might serve as a pathogenic factor for psoriasis, and the blockade of IL-6 might bring benefits to patients with this disease. Most evidence to date, however, suggests otherwise. Tocilizumab, a humanized anti-IL-6 receptor monoclonal antibody, has been approved by the FDA since 2010 for the treatment of moderate-to-severe rheumatoid arthritis (RA). However, in some cases, tocilizumab appeared ineffective for psoriatic arthritis and even induced or exacerbated psoriasis;^155^ this might be explained by the absence of IL-6 leading to excessive production of additional proinflammatory cytokines by keratinocytes.^156^ These findings may provide a new insight into why the blockage of IL-6 was ineffective in the treatment of psoriasis and why some patients with RA treated with IL-6 inhibitors had newly onset psoriasis.

#### IL-9

There are numerous other cytokines and pathways acting as a promoter in the pathogenesis of psoriasis, such as IL-9. The level of IL-9 has been found to be increased in both the serum and the lesions of patients with psoriasis and positively correlated with the severity of psoriasis and impaired quality of life (QoL).^157^ Cells producing IL-9 (the Th9 cells) are also found increased in the skin lesions of psoriasis.^158^ IL-9 can promote the secretion of IL-17, IL-13, IFN-γ, and TNF-α in psoriasis.^158^ However, the mechanisms of how IL-9 regulates the microenvironment of psoriasis still need further research.

#### IL-33

IL-33, also known as “alarmin” and belonging to the IL-1 family, is also reported to be increased in psoriatic lesions.^159^ IL-33 is mainly produced by non-hematopoietic cells such as keratinocytes and fibroblast cells, but is also expressed by hematopoietic cells, especially mast cells (MCs) and DCs.^160^ In psoriasis, MCs can be activated by IL-1 and then produce TNF and IL-33, the latter in turn activating MCs in psoriatic inflammation.^161^ The secreted IL-33 also binds to its receptor, ST2 receptor, to promote the proliferation of keratinocytes, which aggravates the psoriasis. Interestingly, more and more findings have suggested that IL-33 exerts an immunosuppression effect by inhibiting the differentiation and function of Th17 cells.^162^ Hence, more studies are needed to explore the comprehensive role of IL-33 in psoriasis.

### Intracellular signaling pathways of transmission

#### The NF-κB pathway

One of the features of psoriasis is the elevated level of active, phosphorylated nuclear factor kappa B (NF-κB).^128^ NF-κB, a protein transcription factor, is a key regulatory element involved in a variety of immune and inflammatory pathways. The NF-κB pathway plays a crucial role in psoriasis, as it was found to alter the behaviors of keratinocytes and immune cells by affecting their proliferation, differentiation, and production of cytokines or chemokines. NF-κB and its related pathways are also found to be predictive of the patient’s clinical outcome.^163,164^ NF-κB is activated through two pathways: the canonical and non-canonical pathways. The canonical NF-κB pathway usually responds to a variety of stimuli, including ligands of various cytokine receptors, members of the TNF receptor (TNFR) superfamily, pattern-recognition receptors (PRRs), T-cell receptors (TCR), and B-cell receptors.^165^ The major mechanism for canonical NF-κB activation is the polyubiquitination of the multi-subunit IκB kinase (IKK) complex, which induces degradation of IκBα, resulting in transient nuclear translocation and activation of p50/RelA and p50/c-Rel dimers. The IKK complex consists of two catalytic subunits, IKKα and IKKβ, and a regulatory subunit, NF-κB essential modulator (NEMO, also known as IKKγ).^166,167^ The polyubiquitination of IKK is mainly mediated by tumor necrosis factor-associated factors (TRAFs) or E3-ligases. TRAFs, especially TRAF6, catalyze the ubiquitination of adaptor proteins such as ACT1, leading to the phosphorylation of protein kinases, including IKKs.^168^ It is the canonical NF-κB pathway that plays an important role in psoriasis pathogenesis. With regard to the non-canonical pathway, its activation relies on the phosphorylation of p100, which is induced by NF-κB-inducing kinase (NIK). The non-canonical pathway selectively responds to specific members of the TNF cytokine family, such as CD40 ligand and B-cell activating factor (BAFF).^167,168^

Genomic studies have revealed that numerous genes encoding components in the NF-κB pathway are linked to the development of psoriasis. GWAS have found that c-Rel, a gene encoding one of the members in the NF-κB family, is related to psoriasis susceptibility.^169,170^ Downregulation of c-Rel can inhibit the growth of keratinocytes by affecting their cell cycle progression.^171^ IL-23 is regarded as a direct target of c-Rel, and a decreasing level of c-Rel can also inhibit the production of IL-23.^172^ Thus, the susceptibility gene of c-Rel might be a promising target to treat and prevent psoriasis. TRAF3 interacting protein 2 (TRAF3IP2), which encodes Act1, is identified as a novel susceptibility gene for psoriasis and psoriatic arthritis by another GWAS.^41^ Act1 mediates the activation of NF-κB and its related pathway by interacting with TRAF6, a member of the TRAF family, resulting in increased production of inflammatory factors.^173,174^ Of note, CARD14 (also known as CARMA2), a proinflammatory signaling molecule, is usually expressed in several types of skin cells, such as keratinocytes, dermal γδ T cells, and Langerhans cells.^175,176^ Studies have found that CARD14 is located in the psoriasis susceptibility locus 2 (PSORS2) and detected over 20 variations of CARD14 in psoriasis, indicating its vital role in the pathology of psoriasis.^177–179^ Mounting evidence has suggested that the spontaneous psoriasis-like inflammation is caused by the gain-of-function mutation of CARD14.^180,181^ CARD14 interacts with TRAF6 and ACT1, leading to constitutive activation of the NF-κB pathway and the subsequent production of several cytokines and chemokines related to psoriasis.^180^ Apart from the activators of NF-κB, several genes involved in the inhibition of NF-kB pathway were also found to be associated with psoriasis. Among them, NFKBIA, which encodes the NF-κB inhibitor IκB, was identified as a psoriasis susceptibility locus in 2010.^169^ Tumor Necrosis Factor Alpha-Induced Protein 3 (TNFAIP3, also known as A20) and TNFAIP3 Interacting Protein 1 (TNIP1) are also two of the first discovered genes associated with psoriasis.^182^ TNFAIP3 usually works together with TNIP1 to inhibit the NF-kB pathway by inhibiting the polyubiquitination of NEMO (also known as IKKγ), thereby preventing the degradation of the inhibitor molecule IκB.^183,184^ In addition, TNIP1 blocks the conversion of p105 to NF-κB subunit p50, leading to the decrease of NF-κB.^184^ ZC3H12C (encoding MCPIP3) also plays an important role in suppressing NF-κB signaling. A published GWAS has identified an SNP in ZC3H12C that is highly correlated with psoriasis.^178^ It is revealed that ZC3H12C can decrease TNFα-induced IKKα/β, IκBα phosphorylation, and p65 nuclear translocation.^185,186^

### The JAK-STAT and TYK2 pathways

The Janus kinase-signal transducer and activator (JAK-STAT) of the transcription pathway plays a vital role in the intracellular cytokine signaling of a variety of cellular processes, and both of them are mediated by the immune system in normal and pathological states. The JAK family consists of 4 members, namely JAK1, JAK2, JAK3, and tyrosine kinase 2 (TYK2).^187^ JAKs bind to type I and II cytokine receptors to transmit extracellular cytokine signals into cells to activate the STATs, leading to proinflammatory cellular immune responses.^188^ JAKs mediate the signaling of IL-12, IL23, IFN-α, IFN-β and IFN-γ, IL-6 and IL-22 and other cytokines.^188^ Among the JAKs, TYK2 mediates the signaling downstream of IL-23, IL-12, and type I IFN receptors.^189^ The cytokines bind to their receptors, activating receptor-associated JAKs. The activated JAKs can phosphorylate the STAT proteins, leading to STAT dimerize (mostly heterodimers) and translocation to the nucleus to regulate gene transcription.^190^

As discussed above, numerous major pathogenic mediators of psoriasis are linked to the JAK-STAT signaling pathway. JAK1 is directly related to the severity of psoriasis,^191^ and the deletion of TYK2 can suppress psoriasis phenotypes in different mouse models, suggesting their potential therapeutic value for psoriasis.^192^ JAK1, JAK2, and TYK2 are primarily involved in psoriasis due to their presence in all tissues. All three forms of JAKs activate STAT3, resulting in the activation and differentiation of Th17 cells. The JAK1/JAK2-dependent process also participates in the phosphorylation of STAT1, which leads to the activation of IFN-α/β.^193^ The increased expression of STAT1 and STAT3 was also found in psoriatic skin lesions compared to normal skin.^194,195^ More interestingly, genetic linkage studies have revealed an association between TYK2 mutations and psoriasis susceptibility.^196^

### The MAPK pathway

Mitogen-activated protein kinases (MAPKs) are a family of highly conserved serine-threonine protein kinases that engage in signal transmission through a three-level cascade. The MAPKs family includes p38 MAPK, extracellular signal-regulated kinase (ERK), and c-Jun NH2-terminal kinase (JNK).^197^ Each MAPK signaling pathway comprises at least three components, including a MAPK kinase kinase (MAPKKK), a MAPK kinase (MAPKK), and a MAPK. MAPKKKs phosphorylate and activate MAPKKs, which further phosphorylate and activate MAPKs.^198^ It has been demonstrated that ERK1/2, p38, and JNK MAPK are activated and increased in psoriatic lesions, indicating that the MAPK pathway is involved in the pathogenesis of psoriasis.^199–201^

Each MAPK pathway plays an important role in psoriasis. Many signals (e.g., DAMPs, CCN1, and IL-22) in psoriasis can activate the JNK pathway in keratinocytes. The activated JNK pathway in keratinocytes can mediate the recruitment of immune cells in psoriasis by regulating the production of inflammatory cytokines/chemokines, such as IL-6, IL-8, IL-23, IFNγ and TNFα, and CCL20 and hβD-2. In addition to the influence on the proliferation and differentiation of keratinocytes, this activated pathway is also involved in the recruitment and activation of Th1 or Th17 cells to produce additional cytokines such as IL-17, IL-22, and hβD-2.^202,203^ JNK is also recognized as a key factor regulating FOXP3, thereby moderating the development and maturation of Tregs.^204^ It should be noted that, apart from the NF-κB signaling, CARD14 also activates the JNK and p38 MAPK pathways and induces the production of inflammatory cytokines.^205^

The P38 MAPK pathway also plays a crucial role in psoriasis. It has been found that the production of S100A8 induced by the IL-17 and TNF-α is mainly regulated by a P38-dependent mechanism.^206^ Similarly, the production of hBD-2, hBD-3, and S100A7 induced by TNF-α also relies on the phosphorylation of p38 MAPK in keratinocytes.^207^ CCN1 can promote the production of IL-1β in keratinocytes by activating the p38 MAPK signaling.^208^ Mitogen- and stress-activated protein kinase 1 (MSK1) is a downstream target of both p38 and ERK1/2 MAPKs. MSK1 regulates the expression of proinflammatory cytokine genes by activating transcription factors. A prior study found an increased level of phosphorylated MSK1 (Ser376) in psoriatic lesions.^209^ In psoriasis, a high level of IL-6 can lead to increased activation of p-ERK1/2.^210^ Keratin16 (KRT16), a hyperproliferation-associated keratin in keratinocytes, has proven to enhance keratinocyte proliferation and VEGF secretion by activating and phosphorylating the ERK signaling pathway.^211^ Furthermore, DUSP1/MKP-1, a negative regulator of the MAPK pathway, is found to be downregulated in psoriasis. The overexpression of DUSP1 can markedly inhibit keratinocyte proliferation and promote apoptosis by targeting the ERK signaling pathway.^212^

### The PI3K-AKT pathway

The PI3K/Akt pathway has been regarded as an important mechanism in the occurrence and progression of psoriasis.^213,214^ A growing body of evidence has revealed that the activation of this pathway by external or internal stimulation can lead to epidermal hyperplasia, immune pathogenesis, angiogenesis, and other physiological or pathological processes related to psoriasis.^215,216^ Once activated by GFR tyrosine kinase, PI3K converts PIP2 into PIP3 on the plasma membrane. PIP3 binds to the pleckstrin homology (PH) domain of Akt, which enables phosphoinositide-dependent kinase 1 (PDK1) to phosphorylate Thr308 of Akt and PDK2 to phosphorylate Ser473. Once completely activated, Akt acts as the core molecule and moves to the cytoplasm and nucleus to regulate the pathogenesis and progression of psoriasis via the downstream signaling pathway.^217^ As downstream factors of Akt, both forkhead box O (FOXO) and mammalian target of rapamycin (mTOR) regulate the growth, survival, and proliferation of keratinocytes.^218^

FOXO transcription factors are negatively regulated by phosphorylated Akt, and activated FOXO can inhibit cellular proliferation.^219^ It has been demonstrated that the transcriptional activity of FOXO can be regulated by nuclear translocation.^220^ Mounting evidence has revealed elevated expression and activation of PI3K and AKT in the keratinocytes of psoriatic lesions.^221–223^ Higher expression of PI3K may cause Akt hyperactivity, further promoting the proliferation of keratinocytes through the phosphorylation of FOXO. Prior studies also found that FOXO is mainly expressed in the cytoplasm of psoriatic keratinocytes, while is present in the nucleus of skin cells unaffected by psoriasis and normal skin cells.^224^ Hence, excessive activation of P-Akt may change the location of FOXO from the nucleus to the cytoplasm, resulting in reduced inhibition of cell proliferation and hyperproliferation of keratinocytes.

MTOR includes two complexes, mTOR complex 1 (mTORC1) and mTORC2, both of which are essential for the progression of psoriasis due to their function of regulating cell proliferation and inflammation. Activated mTORC1 signaling can be detected in all epidermal layers in psoriasis. A series of cytokines, such as IL-1β, TNF-α, and IL-17A, or a combination of them, have proven to activate the mTORC1 signaling axis.^223^ IL-22 can also activate the Akt/mTOR pathway, promoting the growth of keratinocytes.^225^ Furthermore, studies have shown that the PI3K/Akt/mTOR pathway can regulate both innate and adaptive immune responses. This pathway is believed to be able to moderate the Th1/Th2/Th17 imbalance in psoriasis.^215^ After being activated by cytokines, mTOR is also involved in the secretion of proinflammatory molecules, such as CXCL8, IL-6, and VEGF, by keratinocytes.^226^ It has been suggested that mTORC2 might be essential for FOXP3 stability mediated by chemokine CCL3.^227^ Notably, overactivated mTORC1 contributes to parakeratosis (nuclei retention), which is a form of pathological change of psoriasis.^228^ In brief, the mTOR axis serves as a crucial regulator of inflammation and cell proliferation in psoriasis.

PTEN (phosphatase and tensin homolog deleted on chromosome 10) is a well-known tumor suppressor gene mutated in many human tumors.^229^ It is also a crucial upstream inhibitor of the PI3K/Akt pathway and has been recognized as a FOXO target gene since FOXOs can enhance PTEN transcription.^217^ Accumulating evidence has revealed that the expression of PTEN is reduced in psoriatic lesions, which might be a reason for the hyperproliferation of psoriatic keratinocytes.^230^ In psoriasis, the expression of PTEN can be regulated to adjust its effect on keratinocyte proliferation by a variety of factors, especially miR-RNA such as miR-233, miR-155, and miR-1228-3p.^231–233^

### Other intracellular signaling pathway

In as early as 2010, it was indicated that altered Wnt signaling played an important role in psoriasis, as Wnt-5a was upregulated in psoriatic lesions, whereas WIF-1 was downregulated. A variety of cytokines, such as TGF-α, TNF-α, IFN-γ, and IL-1α, can upregulate the expression of Wnt-5a.^234^ Subsequent studies have found that Wnt-5a is a key regulator of keratinocyte proliferation in psoriasis.^235^ A recent study on the differential DNA methylation of miRNA-encoding genes in psoriasis highlighted the Wnt pathway. The comprehensive involvement of the Wnt pathway indicates that Wnt-mediated signaling not only contributes to the inflammatory condition of psoriasis but also underlies the disease susceptibility.^236^ However, further work on how the Wnt pathway participates in the inflammation and disease susceptibility of psoriasis is warranted.

Moreover, recent studies have identified a novel proinflammatory signaling pathway driven by cyclin-dependent kinase 4 (CDK4)/CDK6 and the methyltransferase EZH2.^237^ CDK4/6 phosphorylates EZH2 in keratinocytes, leading to a methylation-induced activation of STAT3. The activated STAT3 further increases the production of IκBζ, which is a key proinflammatory transcription factor required for cytokine synthesis and is induced in epithelial cells such as keratinocytes by IL-17 and polymorphisms in NFKBIZ (which encodes IκBζ and is recognized as one of the major susceptibility loci for psoriasis).^238^ Thus, treatment targeting the CDK4/6-EZH2 pathway is a promising strategy to tackle psoriasis.

### Crosstalk between different pathways

**Fig. 3:**
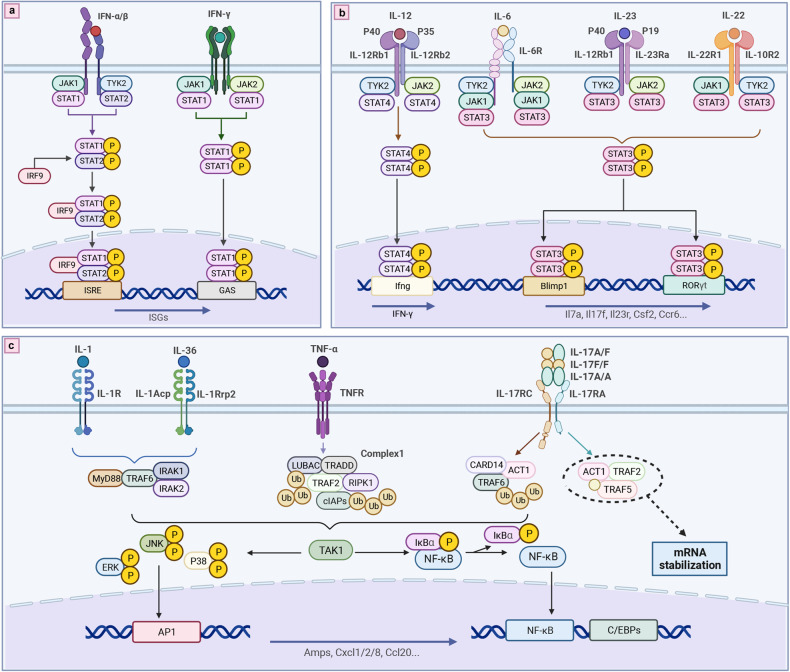
The comprehensive view of intracellular transmission pathway in psoriasis. There are mainly two kinds of transduction way in the psoriatic cytokine. **a**, **b** The one relies on JAK-STAT and TYK2 pathways to regulate gene transcription. Specifically, IFN-α and IFN-β activate STAT1/STAT2 via JAK1 and TYK2, while IFN-γ signaling activates STAT1/STAT1 dimerize via JAK1 and JAK2.The signal of IL-12 activates STAT4/STAT4 dimerize, while IL-6, IL-23, IL-22 activates STAT3/STAT3 dimerize. **c** The other relies on the different cytoplasmic complexes assemble to activate NF-κB/MAPK pathway. The transduction of TNF-α, IL-17, IL-1, and IL-36 is mainly via this. Created with BioRender.com. Abbreviations: IRF9 interferon regulatory factor 9, ISRE interferon-sensitive response element, GAS IFN-γ-activated site, ISGs IFN-stimulated genes, Blimp1 B lymphocyte induced maturation protein 1, RORγt retinoic acid receptor-related orphan receptor gamma t, MyD88 myeloid differentiation factor 88, IRAK interleukin-1 receptor-associated kinases, AP1 activating protein-1, TRAF TNF receptor associated factor, Ub ubiquitin, LUBAC linear ubiquitin chain assembly complex, TRADD TNFR1-associated death domain protein, RIPK1 receptor-interacting serine/threonine-protein kinase 1, cIAP1 cellular inhibitor of apoptosis protein 1, TAK1 TGFβ-activated kinase 1

In psoriasis, cytokines act on their target cells by binding to related membrane receptors to activate complex intracellular pathways. Among all the pathways, the NF-κB pathway, the MAPK pathway, and the JAK-STAT pathway are the most prominent ones to mediate cytokine signal transmissions across the cell membrane, while other pathways are often involved in some other STIM transmissions in psoriasis, including non-coding RNA (Fig. 3).

As the central molecule in the immune microenvironment of psoriasis, IL-17A/IL17A, IL-17F/IL-17F homodimers, and IL-17A/IL-17F heterodimers mediate the ligation in initial subcellular events of IL-17RA/RC and downstream signals by recruiting and activating ACT1, TRAF6, and CARD14 complexes.^180^ TRAF6 further activates the NF-κB pathway by activating IKK, consequently leading to proteasomal degradation of phosphorylated IκB. In psoriasis, nuclear translocation of NF-κB and transcription of NF-κB target genes, such as AP-1 and C/EBPs, are increased.^239^ In addition to activating the NF-κB pathway, the signals can also activate the MAPK pathway (including JNK, ERK, and p38), and the released MAPKs phosphorylate and activate AP1.^240^ Of note, IL-17RA/RC can also recruit other signaling molecules, such as TRAF2 and TRAF5. Unlike TRAF6, TRAF2 and TRAF5 activate RNA-binding proteins rather than enhancing gene transcription, thus promoting mRNA stabilization.^61^

The JAK-STAT and TYK2 pathways play an important role in the signal transmission of cytokines in psoriasis, with different combinations of JAKs mediating different cytokine signaling. JAK2 couples with TYK2 to mediate the signal transduction downstream of IL-12 and IL-23 receptors. IL-12 binds to the IL-12 receptor to activate JAK1 and TYK2, further leading to STAT4 activation, thereby inducing the Th1 cell response. However, IL-23 usually activates STAT3 in Th17 cells, leading to subsequent induction of cytokines such as IL-17A and IL-17F.^33,189^ STAT3 also plays an important role in Th17 cell differentiation and keratinocyte proliferation via JAK1/JAK2 or JAK1/TYK2 signaling induced by IL-6.^241^ The phosphorylation and dimerization of STAT3 by IL-6 and IL-23 signaling enhance the expression and nuclear translocation of RORγt, further activating Th17 gene promoters, including Il17a, Il17f, Il23r, Csf-2, and Ccr6. Moreover, IL-23 signaling-induced transcription factor Blimp-1 enhances the function of Th17 by co-localizing RORγt and STAT-3 at the sites of Il17a, Il23r, and Csf-2 enhancers.^242^ In addition, IL-22 is also responsible for the hyperplasia and differentiation of keratinocytes and the production of anti-microbial peptides by activating STAT3 mediated by JAK1 and TYK2.^146,243^ STAT1 is responsible for the signal transduction of both type I and type II IFNs. IFN-α and IFN-β bind to their receptors, activating STAT1 via JAK1 and TYK2, while IFN-γ signaling exerts effects via JAK1 and JAK2.^244^ Thus, increased STAT1 can result in the production of numerous proinflammatory mediators and the activation and maturation of dendritic cells, which subsequently stimulates Th1 and Th17 cells.^245^

Homotrimers of TNF-α bind to two forms of TNFRs to induce intracellular signaling mainly by recruiting and assembling different cytoplasmic complexes, leading to the activation of different pathways and patient outcomes (including inflammation, cell apoptosis and survival, and tissue regeneration and host defense).^246^ In psoriasis, TNF-α mainly binds to TNFRI to activate downstream signals through complex I, which consists of TNFR1-associated death domain protein (TRADD), TRAF2, receptor-interacting serine/threonine-protein kinase 1 (RIPK1), cellular inhibitor of apoptosis protein 1 (cIAP1) or cIAP2, and linear ubiquitin chain assembly complex (LUBAC).^247^ Once activated, complex I recruits the transforming growth factor-beta (TGFβ) -activated kinase 1 (TAK1) to activate MAPKs, resulting in the activation of the transcription factor AP1; this complex also activates IKK via Lys63-linked ubiquitin, leading to the activation of the downstream NF-κB pathway and the activation of the transcription factor NF-κB.^246,247^ Both transcription factors can regulate proinflammatory gene transcription and immune cell proliferation.

As mentioned above, IL-36 acts on its target cells mainly through IL-1RAcP or IL-1Rrp2, while IL-1β acts on its targets mainly via IL-1R1. It has been found that IL-36γ significantly enhances the interaction between IL-1Rrp2 and the accessory protein IL-1RAcP, subsequently activating the intracellular signaling (including IKBζ/NF-κB, MAPKs, c-Jun, and STAT3 signaling) via the MyD88/IRAK1/IRAK2/TRAF6 mechanism;^112,248^ this is also the primary mechanism by which IL-1β activates MAPKs and NF-κB.^112^

### Metabolites in the pathogenesis of psoriasis

**Fig. 4:**
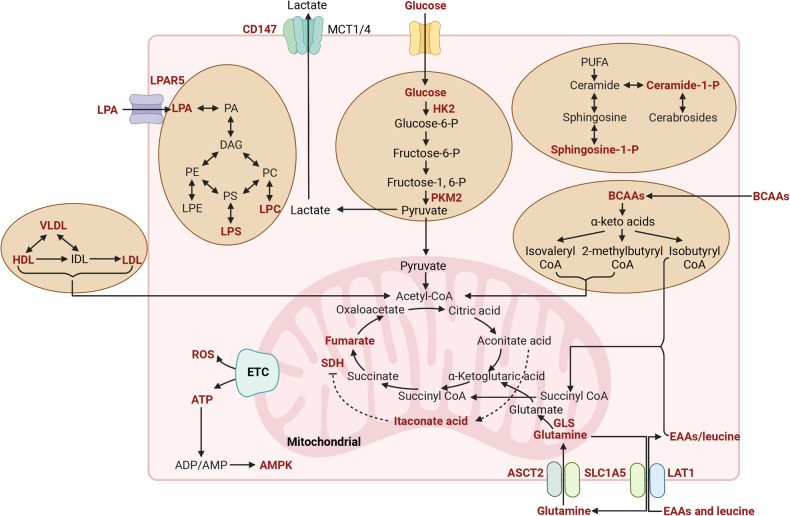
Metabolites in the pathogenesis of psoriasis. Compilation of the main disrupted metabolites and metabolic enzymes (red color) in psoriasis and their interconnections. Created with BioRender.com. Abbreviations: PA phosphatidic acid, DAG diacylglycerol, PE phosphatidylethanolamine, PC phosphatidylcholine, PS Phosphatidylserine, VLDL very low-density lipoproteins, HDL high-density lipoprotein, IDL intermediate-density lipoproteins, LDL low-density lipoproteins, HK2 hexokinase 2, PKM2 pyruvate kinase M2, PUFA polyunsaturated fatty acids, BCAAs branched-chain amino acids, SDH succinate dehydrogenase, GLS glutaminase, EAAs essential amino acid, ETC electron transport chain, AMPK AMP-activated protein kinase, ATP adenosine triphosphate, ADP adenosine diphosphate, AMP adenosine monophosphate, ROS reactive oxygen species

Keratinocyte hyperproliferation is one of the hallmarks of psoriasis. It requires extensive energy-supplying substances such as glucose and maybe the main driver of metabolic changes in psoriasis.^128,249^ As psoriasis has been regarded as a systemic disease related to metabolic abnormalities, researchers strived to investigate the mechanism of how metabolic factors affect the physiology and pathophysiology of psoriasis holistically and systematically. With the advances in metabolomic and bioinformatic analyses, metabolism has been identified as an important factor for the pathogenesis of psoriasis. Multiple forms of cellular metabolism, such as glycolysis, tricarboxylic acid (TCA) cycle, lipid metabolism, and amino acid metabolism, participate in the regulation of keratinocyte as well as related immune cells (Fig. 4). Treatment targeting metabolic factors may also be a potential strategy for coping with psoriasis.^250^

### Metabolites of glycolysis

Glucose is the primary bioenergy source for rapidly proliferating cells, accelerating glycolysis and the TCA cycle. Recent studies found that pro-inflammatory cytokines can stimulate glucose uptake and glycolysis in human keratinocytes.^251^ Glucose uptake involves glucose transporters.^252^ Studies have suggested that the expression of glucose transporter-1 (GLUT-1) is upregulated in psoriatic lesions and is correlated with the severity of disease, indicating the important role of GLUT-1 in psoriasis.^253,254^ Further studies suggest that GLUT-1 is required for the proliferation and stress responses of keratinocytes. Its important role is demonstrated by an animal study, which has shown that specific genetic inhibition of GLUT-1 in keratinocytes can decrease psoriasiform hyperplasia in an IMQ-induced psoriasis-like mouse model. Moreover, topical application of GLUT inhibitor can also alleviate different forms of psoriasis in animal models, indicating that glucose transport might be a promising therapeutic target of psoriasis.^255^ Lately, our group found that HIF-1 α could promote glycolysis, which also plays a crucial role in psoriasis, by upregulating CD147/Basigin and GLUT1.^256^ Pyruvate kinase M2 (PKM2), a key rate-limiting enzyme of glycolysis, has a high level of expression in psoriasis. EGF and IL-17 may both contribute to the upregulation of PKM2.^257,258^ It has been demonstrated that PKM2 is essential for the proliferation of keratinocytes. Conditional PKM2 knockout in keratinocytes could greatly reduce the severity of skin lesions in an IMQ-induced psoriasis-like mouse model.^257^ Recent studies have found that the effect of PKM2 on keratinocytes is mainly exerted through a complex formed with PKM2, Act1, and TRAF6. Subsequently, the complex regulates the NF-κB transcriptional signaling downstream of IL-17 signaling.^258^ In addition to acting on keratinocytes, PKM2 can also enhance the differentiation of Th17 cells, a crucial pathogenetic cell in psoriasis, by activating STAT3 or HIF-1α.^259,260^ All the above evidence suggests that PKM2 might be a potent therapeutic target for psoriasis. As a product of glycolysis, lactate (or lactate acid) modulates immune responses in inflammatory and tumor microenvironments.^261^ Lactate dehydrogenase (LDH) mediates the production of lactate.^262^ The lactate is mainly transported into the extracellular space by two symporters, MCT1 and MCT4, with the aid of CD147.^263^ CD147, an integral transmembrane protein in the immunoglobulin superfamily, can regulate glycolysis associated with MCT1/MCT4 in cancer cells and T cells^263,264^; CD147 also regulates the MCT1 expression and lactate export.^265^ Watanabe et al. found that the serum LDH level might be an indicator of treatment preference, as there was a correlation between clinical improvement in patients treated with apremilast but not in those treated with biologics. However, the serum LDH level was not correlated with the severity of cutaneous disease.^266^ Whether LDH can be a biomarker for psoriasis prediction or efficacy prediction needs more research to explore. Evidence also suggests a significant role of CD147 in the pathogenesis of psoriasis. As early as 2010, our team found elevated CD147 on neutrophils in the peripheral blood of patients with psoriasis, which induced neutrophil chemotaxis^267^; over the next year, we found that CD147 was a molecular marker of high proliferation and low differentiation of keratinocytes. And CD147 might be a psoriasis susceptibility gene. Our further studies demonstrated that the upregulation of CD147 was stimulated by IL-22 via the activation of STAT3 and that CD147 knockdown could reduce the psoriatic changes, such as the production of cytokines, chemokines, and antimicrobial factors induced by IL-22.^268^ As discussed above, Th17 cells play a central role in psoriasis pathogenesis, and the energy required for the differentiation of Th17 cells comes from glycolysis. According to a study by Kanekura et al., CD147 plays an essential role in the development of psoriasis through the induction of Th17 cell differentiation,^269^ expanding the pathologic implication of CD147 in psoriasis.

### Metabolites of the TCA cycle

The TCA cycle is a significant pathway for energy metabolism, and the metabolites also play a crucial role in a variety of physiological processes.^270^ Recent studies have shown the important role of the metabolites of the TCA cycle in the pathogenesis of psoriasis. Specifically, metabolites like lactic acid, pyruvic acid, malic acid, and alpha-ketoneglutaric acid were found to increase in the skin of psoriatic mice, while some other metabolites, such as itaconic acid, were found decreasing.^271^ However, the sample size of this study was limited, indicating that further research is needed to verify the exact mechanism. On this basis, a study by Li et al. emphasizes the significance of metabolites of the TCA cycle in the pathogenesis and treatment of psoriasis. According to their study, the TCA cycle is disordered in patients with psoriasis, and the relapse of this disease might be related to the deficiency of the TCA cycle function. Among the affected metabolites, fumaric acid and itaconic acid are regarded as potential biomarkers for the treatment and pathogenesis of psoriasis,^272^ which is in line with previous findings. Fumarate, as an anti-inflammatory factor, has been found to induce the upregulation of HO-1, further impairing IL-23p19 transcription and STAT1 activation to suppress IL-12 and IL-23.^273^ Clinically, dimethyl fumarate, a fumaric acid ester, is applied to treat moderate-to-severe plaque psoriasis.^274^ Itaconate, a derivative of the TCA cycle, has proven to inhibit IκBζ production, which relies on ATF3.^275^ Itaconate is also regarded as an endogenous SDH inhibitor, while SDH enzyme plays a critical role in the activation and function of human T cells.^276^ Importantly, dimethyl itaconate can markedly alleviate psoriatic-like changes in imiquimod-induced psoriasis-like murine model.^275^ Taken together, the disorder of the TCA cycle plays an important role in the pathogenesis and recurrence of psoriasis, and the intervention on metabolites of the TCA cycle may be a potential strategy to cure psoriasis. However, there are still few studies focusing on the role and mechanism of the TCA cycle in psoriasis. Thus, further research is needed.

### Metabolites of amino acid metabolism

The dysregulation of amino acid metabolism, especially branched-chain amino acid (BCAA) and glutamine metabolism, is also believed to be involved in psoriasis pathogenesis. As early as 2015, Wheelock et al. found that the severity of psoriasis was significantly related to the level of circulating amino acids. Etanercept can also reverse the distinct psoriatic metabotype to a healthy level, indicating that the monitoring of metabolic indicators can help to predict patient response to therapies.^277^ In 2021, our group found that the level of some amino acids, especially essential amino acids (EAAs) and BCAAs, are significantly altered in patients with psoriasis; to be specific, these amino acids included aspartic acid, glutamate, EAAs, BCAAs (leucine, isoleucine, and valine), ornithine, phosphoserine (Pser), other hydrophobic amino acids such as alanine and proline, and aromatic amino acids (AAAs, e.g., phenylalanine and tyrosine).^278^ These amino acids are also related to metabolic diseases such as obesity and insulin resistance, both of which are associated with psoriasis. These findings indicate that amino acid metabolism plays a crucial role in the pathogenesis of psoriasis. However, further studies are still needed to elucidate whether elevated EAAs, BCAAs, and glutamate and downregulated glutamine can predict the risk of psoriasis as well as to investigate the underlying mechanism. In addition, glutamine, the most abundant amino acid in the bloodstream, is also regarded as an important substance with altered levels of psoriasis. Glutamine is an important metabolic fuel, which helps cells rapidly proliferate to meet the increased demand for ATP and biosynthetic precursors. It is transported into cells via the transporter, ASCT2/SLC1A5, and then converted to glutamate in the mitochondria through deamination catalyzed by glutaminase (GLS).^279^ Prior metabolomic analyses have revealed an increased glutamate metabolism in patients with psoriasis, which is positively correlated with the PASI score.^277^ It is also found that glutamine is important for the differentiation of naïve CD4+ T cells to Th17 cells, and that ASCT2/SLC1A5 is necessary for the production of Th1 and Th17 cells and inflammatory T cell responses.^280^ In addition, GLS1-mediated glutaminolysis, is also found to be related to psoriasis pathogenesis. With regard to the mechanism, GLS-1 can promote Th17 and γδ T17 cell differentiation by enhancing the acetylation of histone 3 induced by acetyl-CoA in the Il-17a promoter. Furthermore, GLS1-mediated glutaminolysis can also enhance the proliferation of keratinocytes and the release of chemokines.^281^ This provides a new insight into the link between metabolism and inflammation in psoriasis and indicates that GLS-1 may be a therapeutic target for psoriasis. Interestingly, SLC7A5, also known as LAT1 and being a mediator for the uptake of large neutral amino acids has increased transcriptional levels in psoriatic lesions.^282^ Cibrian et al. further found increased expression of LAT1 protein in keratinocytes and infiltration of lymphocytes in psoriatic lesions. Thus, targeting LAT1-mediated amino acid uptake may also be a useful strategy to control skin inflammation by blocking the expansion of γδ T cells and IL-17 secretion by CD4 T cells.^283^

### Metabolites of lipid metabolism

Lipid metabolism involves lipid synthesis and degradation. The former mainly involves the synthesis of structural and functional lipids, such as glycolipids, phospholipids, sphingolipids, and cholesterol.^284^ To date, lipid metabolism has become a major research interest, as lipids play an important role in almost all biological mechanisms, including the formation and maintenance of the skin barrier.^285^ Studies on lipid metabolism in psoriasis started at the beginning of the 20th century due to the changes in cholesterol levels found in patients with psoriasis.^286^ Subsequent studies have found abnormal bioactive lipids in psoriatic lesions.^287,288^ Among them, sphingolipids attracted more attention due to their effect on keratinocyte growth in psoriasis.^289^ Sphingosine has four derivatives, namely sphingosine-1-phosphate (S1P), ceramide-1-phosphate (C1P), ceramide (Cer), and sphingomyelin (SM). To date, there are controversial views about the role of S1P in psoriasis. Flisiak et al. found a lower serum Cer concentration and significantly higher S1P concentration in patients with psoriasis compared to healthy individuals; however, the S1P level was not related to the severity or duration of psoriasis.^290^ It is reported that S1P exerts anti-proliferative and anti-inflammatory effects in psoriatic mouse models.^291^ Further studies showed that elevating S1P by inhibiting S1P lyase could moderate keratinocyte differentiation and reduce cell proliferation, which was followed by amelioration of IMQ-induced psoriasis-like dermatitis.^292^ Nevertheless, Park et al. pointed out that blocking the production of S1P by a sphingosine kinase 1/2 inhibitor could induce IMQ-induced skin lesions and inflammation through the inhibition of Th17 cell differentiation.^293^ More importantly, a phase II trial revealed that oral ponesimod, a functional antagonist of S1P, is effective in the treatment of moderate to severe chronic plaque psoriasis.^294^ It is without doubt that there were some limitations in these studies, such as the short treatment duration and highly selected population. Hence, more evidence is needed to confirm the effect of S1P on psoriasis. Recently, our group found that there was a significantly higher level of C1P and ceramides phosphate (CerP) in patients with psoriasis, with the higher level of CerP suggesting more severe psoriasis. Moreover, C1P was found to promote inflammation in the IMQ-induced psoriasis-like mouse model, and inhibition of C1P production via a ceramide kinase inhibitor could alleviate the psoriasis-like inflammation.^295^ In addition, using an untargeted lipidomics approach, our team found that substances involved in glycerophospholipid metabolism, such as lysophosphatidic acid (LPA), lysophosphatidylcholine (LysoPC), and phosphatidylinositol (PI), were significantly upregulated in the plasma of patients with psoriasis. This discovery sheds new light on the role of lipids in psoriasis.^296^ Our subsequent studies found that LPA could mediate the pathogenesis of psoriasis by activating keratinocytes through LPAR5.^297,298^ In addition to the influence on keratinocytes, lipid metabolism also plays a vital role in immune cells related to psoriasis, such as plasmacytoid dendritic cells, Th17 cells, and macrophages.^293,299–302^ On the basis of the above finding, psoriasis is also regarded as an immunometabolic disease.^303^ In the future, it is worth working hard to validate the predictive value of such lipid metabolites as biomarkers in psoriasis.

In summary, both keratinocytes and immune cells in psoriasis can be characterized by metabolic disruptions. What a pity is there is a lack of comprehensive understanding of the link between immunology and metabolism. Excitingly, nowadays, studies have yielded insightful findings prompting researchers to investigate potential biomarkers, which will offer novel tools for monitoring and managing psoriasis. Moreover, metabolic reprogramming via suppressing of affected metabolic pathways or metabolites and the dietary restoration of metabolic imbalances may be a prominent therapeutic opportunity to achieve long-term management of psoriasis with minimum adverse effects.

### Epigenetic regulation

**Fig. 5:**
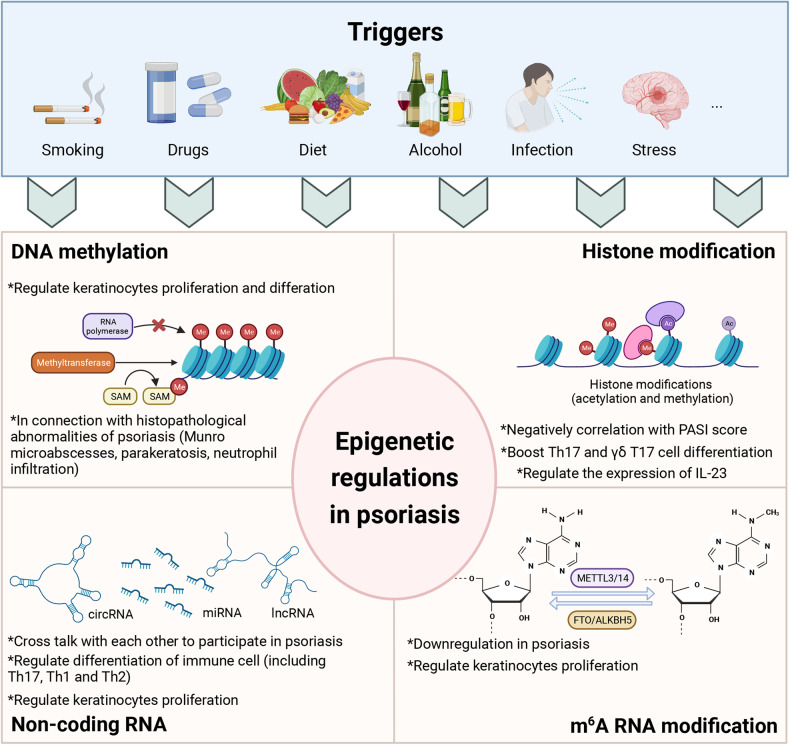
Epigenetic regulations in psoriasis. Environmental triggers such as smoking, medications, diet, alcohol consumption, infection, and stress can alter the expression of genes without changing the DNA sequence. This graph shows the main paradigms of epigenetic modification of psoriasis. Created with BioRender.com

Psoriasis is a chronic and recurrent inflammatory skin disease affected by the complex interplay between genetic and environmental factors,^11^ especially in the presence of the HLA-C*06:02 risk allele, as well as environmental triggers such as infection, stress, smoking, unhealthy diet, medications, and alcohol consumption.^12^ In addition to the genetic differences between monozygotic twins, epigenetic mechanisms have also attracted great attention.^304–306^ Epigenetic modification refers to heritable changes in gene function that take place without any changes in the DNA sequence; this includes DNA methylation, histone modifications, non-coding RNAs, and newly-discovered N^6^-methyladenosine (m^6^A).^307^ To date, epigenetic studies have provided new strategies for the treatment of psoriasis.^308,309^ In this section, we look to present the major epigenetic mechanisms and their roles in the pathogenesis of psoriasis (Fig. 5).

### DNA methylation

DNA methylation is a process to transfer a methyl group from S-adenosylmethionine (SAM) to the 5-carbon position of a cytosine ring catalyzed by DNA methyltransferases (DNMTs), which occurs mainly in CpG dinucleotides.^310^ Research on DNA methylation in psoriasis encompasses two fundamental aspects: genome-wide analysis and the methylation patterns of specific gene loci. Early studies of the epigenetic profile of psoriasis found altered global DNA methylation in psoriatic skin compared to healthy controls.^311–314^ Roberson et al. first identified the methylation of 27,578 CpG sites in skin lesions of patients with psoriasis compared with normal controls; among them, 1108 were differentially methylated, and 12 of these sites were mapped for their epidermal function and differentiation. Moreover, the level of methylations can be reversed after one month of anti-TNF-α treatment.^311^ This observation implies that gene methylation levels may potentially serve as a valuable indicator for monitoring the effectiveness of psoriasis treatment. Zhang et al. further demonstrated that the level of global DNA methylation was elevated in the affected skin and peripheral mononuclear blood cells (PBMCs). They also found a positive correlation between the PASI score and the degree of DNA methylation, rather than the level of PBMC methylation, in the skin lesions of patients with psoriasis.^315^ This indicates that the methylation levels may potentially function as a valuable indicator for clinical diagnosis. However, when methylation of the long interspersed nuclear element-1 (LINE-1) was assessed, hypomethylation was observed in psoriatic skin, with downregulated expression of genes containing LINE-1.^316^ LINE-1 is a retrotransposable element accounting for approximately 20% of the human genome and is commonly used as a surrogate for global methylation.^317^ Nevertheless, the precise mechanism and functional implications of genome-wide methylation in psoriasis remain inadequately elucidated, necessitating further comprehensive investigation.

Zhou et al. observed distinct variations in the methylation levels of these genes using a combination of 262 skin samples and 48 PBMC samples. Notably, genes such as CYP2S1 and S100A8 exhibited such variations.^314^ CYP2S1 encodes a cytochrome P450 protein that is able to upregulate the activity of cAMP-dependent protein kinase in fibroblasts by catalyzing the metabolism of retinoids.^318^ It also inhibits KC proliferation and modulates immune responses through cytokines such as IL-8, IL-33, and CXCL-10.^319^ The S100A family also holds significant sway over the proliferation of KCs and inflammatory response.^320,321^ The study of specific gene methylation patterns can serve as the foundation for subsequent investigations, to identify potential psoriasis susceptibility biomarkers. In addition, it has been found that there is a certain correlation between differentially methylated genes and typical histopathological abnormalities of psoriasis (e.g., Munro microabscesses, parakeratosis, and neutrophil infiltration).^322–324^ However, most of the current studies on DNA methylation in psoriasis used skin tissue or peripheral blood as samples, and cellular heterogeneity might have interfered with the actual influence of epigenetics on psoriasis.

Some studies have suggested that DNA methylation may be a therapeutic target for psoriasis, as some drugs can modulate DNA methylation, affecting the expression of genes involved in inflammation and immune responses.^321,325,326^ Wnt inhibitory factor-1 (WIF1) is a molecule that inhibits the Wnt signaling pathway to regulate cell proliferation and differentiation. It is found that abnormal DNA methylation of WIF1 promoter can reduce its expression, resulting in the proliferation of keratinocytes and the production of IL-8. Treatment with a DNA methylation inhibitor, decitabine, can inhibit the development of psoriasis in IMQ mouse model.^327^ Currently, there are relatively few studies on the treatment of psoriasis that target DNA methylation, but the importance of DNA methylation in psoriasis cannot be ignored due to its implications in the selection of treatments.

### Histone modifications

Histones are structural proteins of chromatin, which form the basic unit of chromatin – nucleosomes with DNA. To date, five types of histones have been found, namely Histone 1 (H1), Histone 2A (H2A), Histone 2B (H2B), Histone 3 (H3), and Histone 4 (H4).^328^ The N-termini of these histones are prone to post-translational modifications (PTM), forming methylation, acetylation, phosphorylation, ubiquitination and other covalent modifications with amino acid residues. In recent years, new types of modifications such as β-hydroxybutyrylation, glycosylation, and lactylation have also been discovered.^329–332^ The histone PTMs can influence gene transcription by inducing local structural changes of chromatin, regulating gene transcription, or by indirectly binding to effect proteins or chromatin remodeling complexes. They can also dynamically regulate the genome by affecting the replication and repair of DNA.^333^ Histone acetyltransferases (HATs) and histone deacetylases (HDACs) are in control of the balance between histone acetylation and deacetylation. Altered expression of the two enzymes has also been found in patients with psoriasis.^334^ Zhang et al. found that the level of H4 histone acetylation in PBMCs of patients with psoriasis was significantly lower than that of healthy controls. And the acetylation level of patients was negatively correlated with the PASI score.^335^ All of the above findings suggest that histone acetylation may play a role in psoriasis. However, it has also been found that the activities of HDAC1, HDAC2, and HDAC3 in psoriatic lesions are not significantly different from those in healthy controls,^336^ which might be related to the different severities of psoriasis in patients included in different groups of the study. The IL-23/IL-17 axis has been identified as a central role in psoriasis. With the advances in research, the histone acetylation of specific genes in this axis has attracted great attention. Xia et al. found that increased histone H3 acetylation of the IL17a promoter could promote Th17 and γδ T17 cell differentiation, which contributed to the immune imbalance and development of psoriasis.^281^ Using a mouse model, Li et al. found that TNF could inhibit both G9A and CLP, which are methyltransferases of H3K9, by altering the level of phosphorylation of related proteins in keratinocytes, thereby decreasing the level of H3K9 methylation and increasing the level of IL-23.^337^ Recently, medications targeting histone modifications have become a hot spot of studies. Vorinostat, an HDAC inhibitor, is found to inhibit KC proliferation to induce their differentiation and apoptosis.^338^ Piperlongumine (PPL) can epigenetically inhibit histone-modifying enzymes, effectively enhancing the interactions of HDAC3 and p65 with IκB, indicating that PPL may be a potential medication in the treatment of psoriasis due to its suppression effect on cell proliferation and inflammation.^339^

In these studies, the abnormal regulation of histone acetylation in psoriasis has been associated with various critical processes, especially inflammatory responses and the proliferation of keratinocytes. This aberrant regulation can induce chromatin relaxation, subsequently modifying the expression of specific genes. Such alterations play a role in driving the development and progression of psoriasis. Up to now, treatment targeting histone modifications is still at the level of cellular and animal models. It is far away to verify its safety and efficacy. It is undeniable that ongoing research efforts will contribute significantly to enhancing our comprehension of this intricate regulatory network, ultimately providing a novel theoretical foundation for the advancement of therapies and treatments for psoriasis.

### Non-coding RNA

With the progress of gene sequencing technology in recent years, the role of non-coding RNA (ncRNA) has attracted great attention as a functional regulatory molecule that mediates a variety of cellular processes, such as chromatin remodeling, transcription, post-transcriptional modification, and signal transduction.^340–343^ There are various types of ncRNAs, including microRNA (miRNA), long noncoding RNA (lncRNA), circular RNA (circ RNA), and circular RNA (circRNA).^344^ With the advance of research, an increasing number of ncRNAs have been found to be associated with the pathophysiology and pathogenesis of psoriasis by regulating the proliferation and differentiation of keratinocytes, secretion of chemokines or cytokines, and activity of T cells.^309,345,346^

MiRNAs are a form of small non-coding RNAs (consisting of around 22 nucleotides). They inhibit gene expression at the post-transcriptional level by specific complementary binding to their target messenger RNAs. A growing body of evidence suggests that miRNAs play an important regulatory role in metabolism, differentiation, inflammation, immunity, and canceration.^347–350^ Altered expression of miRNAs such as miR-210, miR-31, miR-149, miR-125, and miR-155 has been found in skin lesions, peripheral blood mononuclear cells, and plasma of patients with psoriasis.^309,351^ In this section, we look to summarize the pathogenesis of psoriasis related to miRNAs through the influence on a variety of pathways involving immune regulation and keratinocytes.

MiR-210, a hypoxia-associated miRNA, is significantly upregulated in the skin lesions of patients with psoriasis and IMQ-induced mouse models. It is found that miR-210 can promote Th17 and Th1 cell differentiation while inhibiting Th2 cell differentiation through STAT6 and LYN, leading to immune imbalance and inflammatory reactions.^345^ MiR-155 is a central regulatory miRNA that is simultaneously involved in the regulation of multiple genes critical for functional epidermal development.^352^ MiR-155 has been found upregulated in psoriatic skin lesions, LPS-induced keratinocytes, and monocytes^353–357^ and to further regulate psoriatic processes, including the proliferation of psoriatic mesenchymal (PM) stem cells, the release of cytokines or chemokines of psoriatic keratinocytes, and Th17 inflammatory responses. With regard to the mechanism, miR-155 regulates the production of IL-6 and CXCL8 in keratinocytes through the GATA3/IL-37 regulatory axis.^356^ The expression of miR-155 is also increased in CD14^+^ monocytes, which may regulate the proliferation and expression of proinflammatory cytokines as well as the oxidative stress of CD14^+^ monocytes through the TLR4/MyD88/NF-κB signaling pathway.^353^ Moreover, miR-155 may also promote the glycolysis of PM by negatively regulating the TP53INP1/p53 signaling pathway, thereby promoting the proliferation of PM.^357^ MiR-31 has been found upregulated in psoriatic lesions to increase IL-22 expression by targeting periodic tryptophan protein 1 (Pwp1), which is associated with histone modification.^358^ Wang et al. also found that miR-31 could enhance glutamine metabolism and induce Th17 cell differentiation to participate in the development of psoriasis,^359^ which is a new finding on immune-metabolic interaction. However, it is also found that miR-31 is decreased in dermal mesenchymal stem cells (DMSCs), which promote T cell activation by inhibiting the proliferation of DMSCs.^360^ The differences in miR-31 expression in the above studies might have been related to the different cell types studies; thus, the mechanism should still be further explored.

The MiR-125 family can be divided into miR-125a and miR-125b subfamilies.^361^ Raaby et al. found that the expression of miR-125a was downregulated in skin lesions of patients with psoriasis,^362^ and Su et al. also found that miR-125a expression was negatively correlated with the surface area of skin lesions, the Psoriasis Area and Severity Index (PASI) score, the use of phototherapy, and the expression of TNF-α, IL-1β, and IL-17 in patients with psoriasis, which might be indicators for psoriasis. Meanwhile, miR-125a can inhibit KC proliferation and promote apoptosis by negatively regulating the IL-23R/JAK2/STAT3 signaling pathway.^363^ MiR-125b is also found to be downregulated in the skin, plasma, and M5-stimulated KCs in patients with psoriasis, which may affect the proliferation and apoptosis of HaCat cells through the targeted binding to STAT3, BRD4, and SIRT6 and regulate inflammatory responses.^364–366^ Chowdhari et al. found that miR-4516 was downregulated in the skin lesion of patients with psoriasis;^367,368^ miR-4516 binds to STAT3 to inhibit the expression of Bclxl and Cyclin D1^368^ and also targets extracellular matrix protein genes to participate in the differentiation and migration of keratinocytes.^367^ MiR-149 inhibits the production of IL-6, which is induced by TWEAK through the phosphorylation of p38. Furthermore, it is found that high levels of IFN-γ can inhibit the expression of miRNA-149, thereby promoting inflammatory responses in psoriasis.^369^ Other miRNA expression profiles and corresponding targets in psoriasis are presented in Table 1.

**Table 1:** Abnormal microRNAs in psoriasis

MiRNA	Expression status	Tissue/Cells	Binding site	Effect	Reference (PMID number)
miR-203	Upregulated	Psoriatic lesions, HaCaT cells	LXR-α, PPAR-γ	Increase in ki67, KRT5, and KRT14 expression and KC proliferation	32594829
miR-21	Upregulated	Psoriatic lesions	TIMP-3, TACE, ADAM17	Increase in IL-17, IL-21 and IL-23 expression	24574341
miR-146a	Upregulated	Serum	N.A.	N.A.	33937970
		Psoriatic lesions	IRAK1, CARD10	Decrease in serpinb2 expression	31630447
		Psoriatic lesions	FERMT1	Modulated inflammatory responses and KCs proliferation	28595995
miR-31	Downregulated	DMSCs	EMP1, RNF144B, RBMS1, EIG121L	N.A.	30198149
	Upregulated	Psoriatic lesions	PWP1	Increase in IL-22 expression	31278779
		Psoriatic lesions, HaCaT cells	GLUT, GS	Limited glucose availability, elevated glutamine metabolism and induced Th17 differentiation	36855912
miR-210	Upregulated	Psoriatic lesions, peripheral blood CD4^+^ T cells	STAT3, LYN	Promoted Th17 differentiation and inhibited Th2 differentiation through STAT6 and LYN	29757188
miR-340	Downregulated	Psoriatic lesions	IL-17A	Decrease in IL-17A expression	30012847
miR-145-5p	Downregulated	Psoriatic lesions	MLK3	Inhibited KCs proliferation and reduced chemokine secretion	30269330
		PBMCs	N.A.	Inhibited psoriasis progression via the Wnt/β-catenin pathway	34650713
miR-378a-3p	Upregulated	Psoriatic lesions	BMP2	Inhibited KC apoptosis	34244572
miR-155-5p	Upregulated	Psoriatic lesions	CASP3	Inhibited caspase-3 pathway	30904000
miR-617	Upregulated	Psoriatic lesions, HaCaT cells	FOXO4	Promoted proliferation, cell cycle, and reduced apoptosis	33211221
miR-149	Downregulated	Psoriatic lesions	TWEAKR	Suppressed TWEAK signaling and increased inflammatory responses in KCs	33705829
miR-149-5p	Downregulated	Psoriatic lesions	PDE4D	Inhibited IL-22-stimulated KC proliferation	36796259
miR-21-3p	Upregulated	Psoriatic lesions	STAT3, NF-κB	Increased IL-22 expression and promoted KC proliferation	34685526
miR-221-3p	Upregulated	Serum	N.A.	Increase in TNF-α, IL-17 and IL-22 expression	34091460
miR-99a	Downregulated	Psoriatic lesions	FZD5, FZD8	Inhibited HaCaT proliferation through β-catenin and cyclinD1	29441905
		PBMCs	N.A.	N.A.	27562321
		Psoriatic lesions	IGF-1R	Inhibited KCs proliferation and increase in Keratin 10 expression	21687694
miR-369-3p	Upregulated	Psoriatic lesions, serum	N.A.	N.A.	24135466
miR-125b	Downregulated	Psoriatic lesions	STAT3	Promoted SH3PXD2A-AS1 expression by regulatory feedback	33994260
		Serum	BRD4	Activated Jagged-1/Notch signaling pathway	31059052
miR-125b-5p	Downregulated	Psoriatic lesions, M5-stimulated KCs	SIRT6	Inhibited cell growth and inflammation	36592530
		Psoriatic lesions	AKT	Activated Akt/mTOR signaling pathway	31518563
miR-187	Downregulated	Psoriatic lesions	CD276	Increase in IL‐6 expression	30607907
miR-194	Downregulated	Psoriatic lesions	GRHL2	N.A.	28040329
miR-194-5p	Downregulated	HaCaT cells	CDK1	Inhibited proliferation of KCs	34776628
		Psoriatic lesions, HaCaT cells	GAB1	Decrease in Ki67 and MMP9 expression	33393621
miR-4516	Downregulated	Psoriatic lesions	FN1, ITGA9	Suppressed cell motility and proliferation via downregulation of Rac1, RhoA, Cdc42; inhibition of F-actin	28844950
		Psoriatic lesions	STAT3	Decrease in CDK6 and UBE2N expression	24610393
miR-876-5p	Downregulated	Psoriatic lesions, serum	ANG-1	Regulated phosphorylation level of PI3K, AKT, mTOR and ERK	29864894
miR-181b-5p	Upregulated	HaCaT cells	E2F5	N.A.	32801634
	Downregulated	Psoriatic lesions	AKT	Activated AKT/mTOR signaling pathway	31518563
miR-486-3p	Downregulated	Psoriatic lesions	K17	Regulated keratin 17 expression and cell proliferation	28642156
miR-126	Upregulated	HaCaT cells	N.A.	N.A.	33747185
		Psoriatic lesions	N.A.	Decrease in C-caspase and Bcl-2 expression	29943471
	Downregulated	CD4^+^ T cells	N.A.	Regulated Th17 differentiation	34931339
miR-143	Downregulated	PBMCs	N.A.	N.A.	28881648, 24909097
miR-424	Downregulated	Psoriatic lesions	AKT3	Activated AKT/mTOR pathway	34930676
		Psoriatic lesions, serum	MEK1	Inhibited proliferation	21711342
miR-138	Downregulated	CD4^+^ T cells	RUNX3	Increase in the ratio of Th1/Th2	26045321
		Psoriatic lesions	hTERT	Regulated expression of keratin 17 protein	27936398
miR-17-92	Upregulated				27579777
		Psoriatic lesions	CTR9	Deactivation of the STAT3 pathway and release of pro-inflammatory cytokines	35016493
		Psoriatic lesions	CDKN2B, SOCS1	Decrease in CDKN2B, increase in CXCL9 and CXCL10, and regulated STAT1 signaling pathway	29752469
miR-142-3p	Upregulated	M5-stimulated KCs	SEMA3A	Hyper-proliferation and inflammation	32325105
miR-155	Upregulated	CD14^+^ monocytes	N.A.	Regulated TLR4/MyD88/NF-κB signaling pathway	35173453
		Psoriatic lesions, HaCaT cells	N.A.	Activated NLRP3/caspase-1 signaling pathway	29767259
		Psoriatic lesions, LPS-induced keratinocytes	TNFAIP3	Activated IL-17 signaling pathway	33981308
		Psoriatic lesions	GATA3	Decrease in IL37 expression and increase in CXCL8 production	32472715
		Psoriatic lesions	N.A.	Regulated PTEN signaling pathway	28402921
		Psoriatic lesions	TP53INP1	Regulated p53 signaling pathway	35164998
		Psoriatic lesions	N.A.	Decrease in loricrin expression in KCs	33291448
miR-200a	Upregulated	CD4^+^ T cells	N.A.	Leading to immune dysfunction through Th17/Treg cells and relevant cytokines	28738533
miR-200c	Upregulated	Psoriatic lesions, plasma	N.A.	N.A.	31929856
miR-193b	Downregulated	Psoriatic lesions	ERBB4	Regulated STAT3 and NF-κB signaling pathways in KCs	34667159
		Psoriatic lesions, PBMCs	N.A	N.A	25431026
miR-223	Upregulated	Psoriatic lesions, HaCaT cells	PTEN	Modulated PTEN/Akt signaling pathway	31108094
miR-122-5p	Upregulated	HaCaT cells	SPRY2	Regulated extracellular signal kinase and mitogen-activated protein kinase signaling pathway	27943426
		PBMCs	N.A	Regulated MAPK, JAK-STAT, and NF-κB signaling pathways	33975513
miR-20a-3p	Downregulated	Psoriatic lesions, HaCaT cells	SFMBT1	Regulated TGF-β1/survivin signaling pathway in KCs	29886071
miR-383	Downregulated	Psoriatic lesions	LCN	Inhibition of JAK/STAT signaling pathway	33819732

LncRNAs are non-coding RNAs consisting of approximately 200 nucleotides and play an important role in autoimmune diseases, including systemic lupus erythematosus, rheumatoid arthritis, ulcerative colitis, and psoriasis.^370–373^ Using RNA sequencing, Tsoi et al. found that more than 40% of novel lncRNAs were differentially expressed in psoriatic lesions. These lncRNAs were found to be co-expressed with immune-related genes and enriched in epidermal differentiation complexes, suggesting that they might be involved in the pathogenesis of psoriasis.^374^ Through the comparison of skin group between patients with psoriasis and healthy volunteers using microarray technology, Yan et al. found significantly altered expression in 2194 lncRNAs and 1725 mRNAs.^373^ To be specific, the expression of NORAD was found upregulated in IL-22/LPS-stimulated HaCaT cells, possibly through the negative regulation of miR-26a, thereby upregulating the expression of proteins associated with keratinocyte proliferation (e.g., K6, K16, and K17).^375^ MSX2P1, which is highly expressed in psoriatic lesions, promotes keratinocyte growth by inhibiting miR-6731-5p and activating S100A7.^376^ Furthermore, MIR31HG and RP6-65G23.1 can accelerate HaCaT cell proliferation by promoting the cell cycle.^377,378^ Some lncRNAs, such as MEG3 and LOC285194, are significantly downregulated in psoriatic lesions. MEG3 inhibits keratinocyte proliferation by regulating the expression of miR-21 and caspase-8.^379^ LOC285194 negatively regulates the expression of miR-616, which in turn leads to GATA3 activation, ultimately reducing keratinocyte viability.^380^ By comparing patients with psoriasis and individuals with no skin diseases, a study identified 971 differentially expressed lncRNAs and 157 differentially expressed lncRNAs after treatment with adalimumab, highlighting the potential role of lncRNAs in the physiology of psoriasis and patient response to treatment.^381^ The lncRNA TRAF3IP2-AS1 regulates Act1-meditated IL-17A signaling by inhibiting IRF1, a transcription factor of Act1; this might be the reason why patients with psoriasis who have the Act1 (D10N) variant are unlikely to respond well to IL-17A inhibitors.^382^

CircRNAs, a special form of ncRNA molecules generated by alternative splicing, are evolutionarily highly conserved.^383^ It can competitively bind to miRNAs to attenuate the translational repression or degradation of their target gene mRNAs via miRNAs.^384^ Increasing evidence suggests that circRNAs are involved in psoriasis pathogenesis by regulating cell proliferation and inflammation.^385–387^ Qiao et al. found that hsa_circ_0061012 was significantly upregulated in psoriatic lesions and that it might be a candidate biomarker for psoriasis.^388^ In addition, circ_0003738 is significantly up-regulated in Treg cells in psoriasis and may reduce the inhibitory function of Tregs through miR-562/IL-17RA and miR-490-5p/IFNGR2, thereby promoting the inflammatory responses in psoriasis.^389^

In summary, ncRNA-mediated epigenetic regulation is closely related to the development and progression of psoriasis. The ncRNAs detected in blood or urine samples can be used as biomarkers for diagnosis and prognosis. Transport of ncRNAs to skin tissues by modified nucleic acid technology may be a potential alternative treatment for psoriasis. However, high complexity is still a problem in the application of ncRNAs in clinical practice, and the specific effect of each form remains to be further confirmed in more preclinical studies.

### m^6^A methylation

With the development of sequencing technology in recent years, m^6^A has gradually become a hot spot in epigenetic studies. The m^6^A modification refers to the methylation of the nitrogen atom at the 6th site of adenine in RNA molecules. It is found that m^6^A methylation is involved in a variety of biological behaviors of cells, which is a highly specific, dynamic, and reversible biological process that involves three types of enzymes: methylation transfer protein (writer), demethylation protein (eraser) and recognition protein (reader).^390,391^ Using RNA sequencing, Wang et al. found that transcripts of the psoriatic skin sample had the fewest m^6^A peaks and the lowest m^6^A peak density. Besides, the greatest differences in m^6^A methylation were observed in the comparison between psoriatic skin and skin of healthy individuals or between psoriatic skin and unaffected skin of patients with psoriasis. Moreover, the upregulation of gene expression was often accompanied by the upregulation of m^6^A methylation, suggesting a regulatory role of m^6^A in psoriasis.^392^ Oerum et al. found that hsa_circ_0004287 was upregulated in PBMCs of patients with psoriasis. This upregulation appears to be associated with the impact of has_circ_0004287 on reducing the stability of its host gene metastasis associated lung adenocarcinoma transcript 1 (MALAT1) by competitively binding to IGF2BP3 in an m^6^A-dependent manner, which subsequently leads to the phosphorylation of p38/mitogen-activated protein kinase and the initiation of macrophage-mediated inflammation.^346^ Xia et al. also found that AGAP2-AS1 could be upregulated in the skin of patients with psoriasis through the modification of m6A methylation, thereby promoting keratinocyte proliferation through the miR-424-5p/AKT/mTOR axis.^393^ Lately, Xiao et al. concluded that METTL3-mediated m^6^A methylation equilibrated γδ T1 and γδ T17 cells to participate in the pathogenesis of psoriasis.^394^ However, there is still a lack of studies on m^6^A, suggesting the need for more experiments to demonstrate the role of m^6^A in psoriasis.

## Comorbidities of psoriasis and their molecular mechanistic cascades

**Fig. 6:**
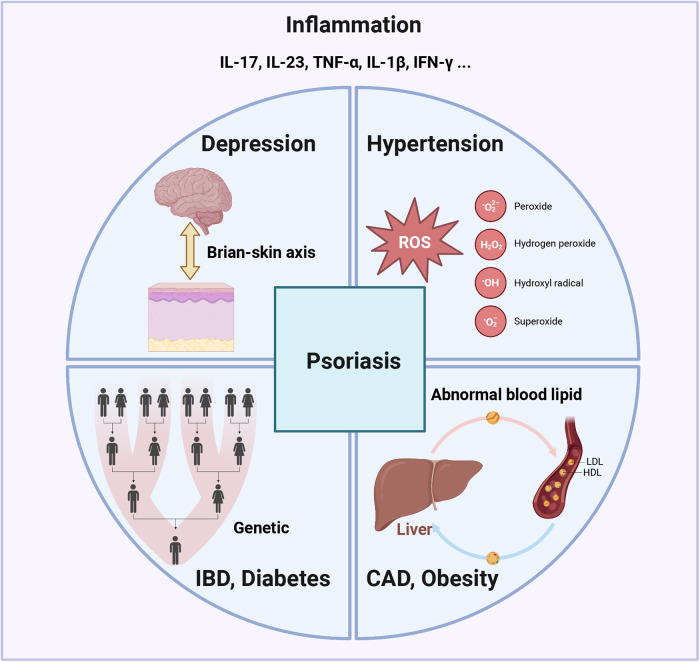
Mechanistic models of psoriasis comorbidities. Chronic inflammatory processes directly lead to psoriatic arthritis, and they contribute to other comorbidities when coexisting with other factors. Driven by genetic factors and increased cytokines, diabetes, and IBD are likely to develop in psoriasis. Dyslipidemia, together with abnormal cytokine levels, can lead to obesity and coronary heart diseases. With a hyperactive HPA axis, psoriatic cytokines can further lead to depression. Created with BioRender.com

The incidence of comorbidities in patients with psoriasis was first reported in 1897, indicating the early awareness of the coexistence of psoriasis and other diseases as well as the early exploration of common pathogenic mechanisms.^395^ Subsequent clinical and epidemiological studies have shown that patients with psoriasis may have multiple comorbidities, including metabolic syndrome (e.g., diabetes, hypertension, dyslipidemia, and obesity), cardiovascular disease, depression, and psoriatic arthritis.^396–398^ Some clinical studies suggested a wider variety of comorbidities of psoriasis, including schizophrenia, asthma, inflammatory bowel disease (IBD), Crohn’s disease, chronic kidney disease, and Grave’s disease.^399^ Furthermore, an increasing number of studies have shown that psoriasis is associated with an increased risk of cancer.^400,401^ The presence of psoriasis comorbidities can also adversely affect the outcomes, quality of life, life expectancy, as well as psychosocial and economic status of patients. A better understanding of the involved signaling pathways and physiological mechanisms can be helpful for treating psoriasis and its comorbidities as early as possible (Fig. 6).

Common genetic background, increased oxidative stress, overlapping chronic inflammatory processes, abnormal immune regulation mechanisms, and the brain-skin axis may be the basis of multiple comorbidities of psoriasis. Among them, the abnormal immunopathophysiological events play a central role.

Prior GWAS have identified several shared genetic loci (including PSORS2, PSORS3, and PSORS4) and mutual genes (including CDKAL1, ST6GAL1, PTPN22, and JAZF1) that link psoriasis and the susceptibility to diabetes.^402,403^ Moreover, the increased key cytokines in psoriasis, such as IL-23, IL-17, IL-1β, IFN-γ, and TNF-α can damage islet β cells in a separate and synergistic manner. For instance, IL-1β can induce insulin resistance by activating the p38MAPK pathway,^404^ and TNF-α mediates insulin resistance by inhibiting the activity of insulin receptors to contribute to the development of diabetes.^405^ These may be the potential molecular mechanisms of the comorbidity of diabetes in patients with psoriasis. Driven by genetic factors and increased cytokines, IBD can also appear as a comorbidity of psoriasis.^406,407^

Although current epidemiological data from cross-sectional and observational studies have suggested a high risk of hypertension in patients with psoriasis, the exact pathophysiological mechanism is still unclear. Possible mechanisms include the release of keratinocyte-derived vasoactive peptides such as endothelin-1.^408^ Excessive endothelin-1 release by hyperplastic keratinocytes of psoriasis can lead to vascular inflammation and endothelial dysfunction via promoting cytokine and ROS production, which has been identified as a primary feature of hypertension pathophysiology.^409,410^

Dyslipidemia is frequently observed in patients with psoriasis; in such patients, decreased levels of high-density lipoprotein (HDL) or increased levels of low-density lipoprotein (LDL), especially oxidative LDL, have been found.^411^ The increased oxidized LDL in psoriasis patients is engulfed by macrophages and forms arterial plaque, leading to cardiovascular diseases such as atherosclerosis and coronary artery disease (CHD).^412^ Moreover, the released IL-17, IL-22, IL-6, and IL-21 in psoriasis can cause plaque instability and hemorrhage.^413^ In addition to CHD, dyslipidemia is also an independent risk factor for comorbidities of psoriasis, such as obesity.^414^ It has been found that a variety of psoriasis-related cytokines, such as TNF-α, IL-1β, and IL-6, can lead to obesity by regulating triglyceride metabolism and adipocyte differentiation.^415^

Among all the comorbidities, depression is the most common and relevant neurological comorbidity in patients with psoriasis.^416^ Numerous studies have shown that mental stress is an essential contributor to psoriasis, and the deterioration of psoriasis can increase the risk of depression and anxiety.^417^ The brain-skin axis is believed to play an essential role in this process.^418,419^ It is revealed that cytokines such as IL-1β, TNF-α, and IL-6, which are increased in psoriasis, can lead to the increase in cortisol by downregulating the negative feedback loop of the HPA system, resulting in hyperactivity of the HPA system and ensuing depression.^420^

## Current progress in targeted therapy for psoriasis

### Overview of therapeutic options

Psoriasis has a high recurrence rate despite a variety of treatment options.^12^ In clinical settings, the aims of treatment for psoriasis include removing skin lesions, reducing itching and other distressing symptoms, and improving patients’ quality of life. It is also necessary to reduce the frequency of psoriasis recurrence and its complications and control adverse reactions during the treatment.^421^ The selection of treatment approaches is based on the accurate assessment of the severity of this disease, which is usually performed using the PASI. The PASI is rated from aspects of the location, area, scaling, infiltration, and erythema of the lesions, with a total score of 0–72.^422^ According to the consensus, mild psoriasis was defined as ‘PASI ≤ 10’, and for moderate-to-severe psoriasis, ‘PASI > 10’.^423^ Apart from PASI, Physician Global Assessment (PGA), Nail Psoriasis Severity Index (NAPSI), Pustular Psoriasis Physician Global Assessment (GPPGA), and American College of Rheumatology criteria (ACR) are also used to assess the severity of different types of psoriasis.^424,425^

Topical corticosteroids, calcipotriol, or their combination are the primary treatment methods for mild psoriasis.^12^ Topical corticosteroids exert anti-inflammatory, antiproliferative, and local vasoconstrictive effects by downregulating genes that encode pro-inflammatory cytokines.^11^ A study found that 40–75% of patients with psoriasis achieved PASI 75 response after using topical corticosteroids.^426^ However, potent corticosteroids cannot be used long-term due to their adverse effects of subcutaneous fat atrophy and adrenal suppression.^427^ Calcipotriol is a vitamin D3 derivative that can inhibit epidermal hyperplasia and induce normal epidermal differentiation in psoriasis.^428^ It has been found that the combined formulation of these two medications, calcipotriol/betamethasone, has better efficacy and remission rate than the monotherapy with either medication, with less irritation and fewer adverse reactions of the skin.^429,430^

Other topical medications for psoriasis include topical calcineurin inhibitors, topical keratolytic agents, and coal tar. Topical calcineurin inhibitors exert a therapeutic effect on psoriasis mainly by acting on calcineurin targets, thereby inhibiting T cell activation. Due to their advantage of not causing facial lipoatrophy, calcineurin inhibitors are often used on the face.^431^ Topical keratolytic agents mainly include tazarotene, which can inhibit keratinocytes, thereby breaking down the thick flakes of psoriatic plaques.^11^ At present, coal tar is rarely used due to its mutagenicity.

Oral medications, such as methotrexate, cyclosporine, and retinoids, are usually used for moderate-to-severe psoriasis that cannot be controlled by topical drugs.^423^ Methotrexate was approved for the treatment of psoriasis more than 50 years ago by the US Food and Drug Administration (FDA). As the most commonly used treatment option before the introduction of biologics, methotrexate has a certain effect on all types of psoriasis. Despite its therapeutic effect, methotrexate has unignorable adverse reactions such as hepatotoxicity and severe gastrointestinal reactions;^432^ therefore, folic acid is often recommended to reduce the side effects during the treatment.^11^ Cyclosporine is also used to treat different types of severe psoriasis. Flytstrom et al. found that cyclosporine is more effective than methotrexate in the treatment of plaque psoriasis. Still, it is also associated with more severe renal toxicity and other adverse reactions such as gastrointestinal discomforts and hirsutism, which might be why it is limited in clinical practice.^433^ Unlike methotrexate and cyclosporine, acitretin, a systemic synthetic retinoid, is usually used to treat erythrodermic psoriasis and pustular psoriasis. However, acitretin cannot be used by pregnant individuals or planning for pregnancy because of its teratogenic effect; other adverse effects include dry skin, dyslipidemia, and elevation of transaminases.^11,434^

Phototherapy is also an important treatment option for moderate to severe psoriasis. There are many types of phototherapies, including psoralen and UV-A (PUVA) (320–400 nm), wide-band ultraviolet B (UVB) (290–320 nm), and narrow-band ultraviolet B (NB-UVB) (311 nm).^435^ NB-UVB has been used as a first-line phototherapy for plaque psoriasis for its better efficacy, longer remission time, and fewer adverse reactions.^435^ It is also found that the combination of acitretin and NV-UVB can achieve better efficacy with fewer adverse reactions in the treatment of plaque psoriasis.^436^

With a deeper understanding of the pathogenesis of psoriasis in the past 20 years, biologics have been gradually used for targeted immunotherapy with promising efficacy and fewer side effects, attracting great attention. Thus, we hereby summarize the targeted therapies for psoriasis (Table 2).

**Table 2:** Targeted therapy of psoriasis

Target	Molecule	Types of psoriasis	Drug dose	Efficacy		PMID
TNFα	Etanercept	plaque psoriasis	50 mg twice weekly	PASI 75	49.0% at week 12	14627786
					59.0% at week 24	
					47.0% at week 12	16399150
		psoriatic arthritis	50 mg once weekly	ACR 20	60.8% at week 12	20124563
					71.7% at week 24	
			50 mg twice weekly	ACR 20	66.4% at week 12	
					69.0% at week 24	
	Infliximab	plaque psoriasis	week 0, 2, and 6, then every 8 week to week 46	PASI 75	80.0% at week 10 82.0% at week 24	16226614
				PASI 90	57.0% at week 10 58.0% at week 24	
			5 mg/kg at week 0, 2, 6, 14 and 22	PASI 75	72.0% at week 24	27416891
					67.0% at week 48	
		psoriatic arthritis	5 mg/kg at week 0, 2, 6, 14 and 22	ACR 20	58.0% at week 14	15677701
	Adalimumab	plaque psoriasis	80 mg at week 0; 40 mg until week 16 twice weekly	PASI 75	71.0% at week 16	17936411
			40 mg once weekly	PASI 75	80.0% at week 12	17010738
			40 mg twice weekly	PASI 75	53.0% at week 12	
		psoriatic arthritis	40 mg twice weekly	ACR 20	74.0% at week 12	19815494
				ACR 50	51.0% at week 12	
				ACR 70	32.0% at week 12	
		nail psoriasis	80 mg at week 0; 40 mg until week 16 twice weekly	NAPSI 75	46.6% at week 26	28993005
IL-17A	secukinumab	plaque psoriasis	150 mg (administered once weekly for 5 week, then every 4 week),	PASI 75	71.6% at week 12 67.0% at week 12	25007392
				PASI 90	39.1% at week 12 41.9% at week 12	
			300 mg (administered once weekly for 5 weeks, then every 4 week),	PASI 75	81.6% at week 12 77.1% at week 12	
				PASI 90	59.2% at week 12 54.2% at week 12	
		psoriatic arthritis	150 mg (once a week from baseline and then every 4 week from week 4)	ACR 20	51.0% at week 24	26135703
				ACR 50	35.0% at week 24	
			300 mg (once a week from baseline and then every 4 week from week 4)	ACR 20	54.0% at week 24	
				ACR 50	35.0% at week 24	
		nail psoriasis	150 mg (once a week from baseline and then every 4 week from week 4)	NAPSI	37.9% at week 16 52.6% at week 32	30367462
			300 mg (once a week from baseline and then every 4 week from week 4)	NAPSI	45.3% at week 16 63.2% at week 32	
	Ixekizumab	plaque psoriasis	160 mg at week 0, and then 80 mg twice weekly	PSAI 75	89.1% at week 12 87.0% at week 12	27299809
				PSAI 90	70.9% at week 12 68.0% at week 12	
				PSAI 100	35.3% at week 12 38.0% at week 12	
			160 mg at week 0, and then 80 mg every four weekly	PSAI 75	82.6% at week 12 84.0% at week 12	
				PSAI 90	64.6% at week 12 65.0% at week 12	
				PSAI 100	33.6% at week 12 35.0% at week 12	
		psoriatic arthritis	160 mg at week 0, and then 80 mg twice weekly	ACR 20	48.0% at week 24	28551073
				ACR 50	33.0% at week 24	
				ACR 70	12.0% at week 24	
			160 mg at week 0, and then 80 mg every four weekly	ACR 20	53.0% at week 24	
				ACR 50	35.0% at week 24	
				ACR 70	22.0% at week 24	
		nail psoriasis	160 mg at week 0, and then 80 mg twice weekly	NAPSI	35.2% at week 12	27910156
				NAPSI 0	17.5% at week 12	
			160 mg at week 0, and then 80 mg every four weekly	NAPSI	36.7% at week 12	
				NAPSI 0	19.7% at week 12	
IL-17F and IL-17A	Bimekizumab	plaque psoriasis	320 mg every 4 week	PSAI 75	95.4% at week 16 93.3% at week 16^a^	33549192 33891380^a^
				PSAI 90	90.8% at week 16 85.5% at week 16^a^	
				PSAI 100	68.2% at week 16 61.7% at week 16^a^	
		psoriatic arthritis	160 mg every 4 week	ACR 20	67.0% at week 16 62.0% at week 16^a^ 65.0% at week 24^a^	36495881 36493791^a^
				ACR 50	43.4% at week 16 44.0% at week 16^a^ 45.0% at week 24^a^	
				ACR 70	26.6% at week 16 24.0% at week 16^a^ 29.0% at week 24^a^	
IL-17 receptor	Brodalumab	plaque psoriasis	210 mg at week 0,1,2,4,6,8,10	PASI 75	86.0% at week 12 85.0% at week 12	26422722
				PASI 100	44.0% at week 12 37.0% at week 12	
			140 mg at week 0,1,2,4,6,8,10	PASI 75	67.0% at week 12 69.0% at week 12	
				PASI 100	26.0% at week 12 27.0% at week 12	
		psoriatic arthritis	280 mg at week 0,1,2,4,6,8,10	ACR 20	39.0% at week 12	24918373
				ACR 50	14.0% at week 12	
				ACR 70	5.0% at week 12	
			140 mg at week 0,1,2,4,6,8,10	ACR 20	37.0% at week 12	
				ACR 50	14.0% at week 12	
				ACR 70	5.0% at week 12	
			210 mg twice weekly	ACR 20	51.8% at week 16 44.3% at week 16	33106286
				ACR 50	28.8% at week 16 23.2% at week 16	
				ACR 70	12.5% at week 16 10.2% at week 16	
			140 mg twice weekly	ACR 20	39.5% at week 16 50.9% at week 16	
				ACR 50	18.3% at week 16 9.3% at week 16	
				ACR 70	7.8% at week 16 13.3% at week 16	
IL12/IL23 p40	Ustekinumab	plaque psoriasis	45 mg (at week 0 and 4 and then every 12 week)	PASI 75	67.1% at week 12 66.7% at week 12^a^ 71.2% at week 28 69.5% at week 28^a^	18486739 18486740^a^
				PASI 90	41.6% at week 12 42.3% at week 12^a^ 49.2% at week 28 44.8% at week 28^a^	
				PASI 100	12.5% at week 12 18.1% at week 12^a^ 20.8% at week 28 18.6% at week 28^a^	
			90 mg (at week 0 and 4 and then every 12 week)	PASI 75	66.4% at week 12 75.7% at week 12^a^ 78.6% at week 28 78.5% at week 28^a^	
				PASI 90	36.7% at week 12 50.9% at week 12^a^ 44.7% at week 28 54.3% at week 28^a^	
				PASI 100	10.9% at week 12 18.2% at week 12^a^ 29.2% at week 28 29.5% at week 28^a^	
		psoriatic arthritis	45 mg (at week 0 and 4 and then every 12 week)	ACR 20	42.4% at week 24	23769296
			90 mg (at week 0 and 4 and then every 12 week)	ACR 20	49.5% at week 24	
		nail psoriasis	45 or 90 mg, according to body weight, at week 0, 4, and 16	NAPSI	46.5% at week 24 (45 mg) 48.7% at week 24 (90 mg)	24117389
IL-23 p19	Guselkumab	plaque psoriasis	100 mg at week 0 and 4 then every 8 weeks	PASI 75	89.0% at week 12	31402114
				PASI 90	69.0% at week 12 76.3% at week 48	
				PASI 100	58.0% at week 48	
		psoriatic arthritis	100 mg at week 0, 4, then every 4 weeks	ACR 20	59.0% at week 24	32178765
			100 mg at week 0, 4, then every 8 weeks	ACR 20	52.0% at week 24	
	Tildrakizumab	plaque psoriasis	100 mg at week 0, 4, 12, 16	PASI 75	64.0% at week 12 61.0% at week 12 80.0% at week 28	28596043
				PASI 90	35.0% at week 12 39.0% at week 12 52.0% at week 28	
				PASI 100	14.0% at week 12 12.0% at week 12 24.0% at week 28	
			200 mg at week 0, 4, 12, 16	PASI 75	62.0% at week 12 66.0% at week 12 82.0% at week 28	
				PASI 90	35.0% at week 12 37.0% at week 12 59.0% at week 28	
				PASI 100	14.0% at week 12 12.0% at week 12 32.0% at week 28	
	Risankizumab	plaque psoriasis	150 mg at week 0, 4, 16, 28, and 40	PASI 90	75.3% at week 16 74.8% at week 16 81.9% at week 52 80.6% at week 52	30097359
				PASI 100	35.9% at week 16 50.7% at week 16 56.3% at week 52 59.5% at week 52	
IL-36 receptor	Spesolimab	Generalized pustular psoriasis	a single intravenous dose of 900 mg	GPPGA 0	54% at week 1	34936739
JAK1, JAK3	Tofacitinib	psoriatic arthritis	5 mg twice daily	ACR 20	54.0% at month 3 73.0% at month 12	29045212
				ACR 50	30.0% at month 3 48.0% at month 12	
			10 mg twice daily	ACR 20	63.0% at month 3 73.0% at month 12	
				ACR 50	42.0% at month 3 50.0% at month 12	
JAK1	Upadacitinib	psoriatic arthritis	15 mg once daily	ACR 20	70.6% at week 12 56.9% at week 12^a^ 73.4% at week 24	33789011 33272960^a^
				ACR 50	37.5% at week 12 52.4% at week 24	
				ACR 70	15.6% at week 12 28.7% at week 24	
			30 mg once daily	ACR 20	78.5% at week 12 63.8% at week 12^a^ 78.5% at week 24	
				ACR 50	51.8% at week 12 60.5% at week 24	
				ACR 70	25.3% at week 12 36.4% at week 24	
TYK2	Deucravacitinib	plaque psoriasis	6 mg once daily	PSAI 75	58.4% at week 12 69.3% at week 24	35820547
				PSAI 90	35.5% at week 12 42.2% at week 24	
				PSAI 100	14.2% at week 12 17.5% at week 24	
PDE4	Apremilast	plaque psoriasis	30 mg twice a day	PSAI 75	33.1% at week 16	26089047
				PSAI 90	9.8% at week 16	
		psoriatic arthritis		ACR 20	40.0% at week 16	24595547
	roflumilast	plaque psoriasis	Cream for once daily	IGA success	37.5% at week 8 42.4% at week 8	36125472

PASI 75: the proportion of patients who had a reduction by ≥75% in Psoriasis Area and Severity Index; PASI 90: the proportion of patients who had a reduction by ≥90% in Psoriasis Area and Severity Index; PASI 100: the proportion of patients who had a 100% reduction in Psoriasis Area and Severity Index

ACR 20: the proportion of patients who had a ≥ 20% improvement according to the American College of Rheumatology criteria; ACR 50: the proportion of patients who had a ≥ 50% improvement according to the American College of Rheumatology criteria; ACR 70: the proportion of patients who had a ≥ 70% improvement according to the American College of Rheumatology criteria

NAPSI 75: the proportion of patients who had ≥75% improvement in total-fingernail modified Nail Psoriasis Severity Index (NAPSI75)

NAPSI: Nail Psoriasis Severity Index

NAPSI 0: the proportion of patients who achieved a NAPSI score of 0

IGA success: Investigator Global Assessment (IGA) is clear or almost clear status plus ≥2-grade improvement from baseline [score range, 0–4]

GPPGA 0: the proportion of patients who scored 0 on the Generalized Pustular Psoriasis Physician Global Assessment pustulation subscale

^a^The results regarding efficacy were obtained from the PMID literature with the same^a^ for the same molecule

### TNFα inhibitors

TNF-α inhibitors are the first biological agents approved for the treatment of moderate-to-severe psoriasis and psoriatic arthritis. At present, three main biologics, i.e., *etanercept*, *infliximab*, and *adalimumab*, are used to treat psoriasis by targeting TNFα to prevent TNF-mediated cellular responses and modulate biological responses that are controlled by additional downstream molecules induced or regulated by TNFs.

Etanercept is a recombinant TNFα receptor fusion protein that can competitively bind to the TNF-α receptor p75 Fc on the cell surface to inhibit TNF-mediated cellular responses.^437^ It is the first TNFα inhibitor approved by the FDA for the treatment of plaque psoriasis and psoriatic arthritis.^438^ Prior clinical studies have found that etanercept can significantly relieve plaque psoriasis and psoriatic arthritis; to be specific, this medication helped achieve a PASI response rate of 47–49% at week 12 of treatment with etanercept and a PASI 75 response rate of 59% at week 24 of treatment at a dose of 50 mg twice weekly;^439,440^ moreover, after 12 weeks of treatment with etanercept for psoriatic arthritis, the ACR 20 reached 66.4–73% with well tolerance.^441,442^

Infliximab is an IgG1 mouse-derived monoclonal antibody specific for TNFα,^443^ which was approved by the FDA for the treatment of psoriatic arthritis and plaque psoriasis in 2005 and 2006, respectively.^435^ Infliximab has prominent effects in treating plaque psoriasis or psoriatic arthritis;^443^ it is reported that infliximab achieved a PASI 75 response rate of 80–88% at week 10 and helped to maintain a PASI 75 response rate of 72–82% at week 24 when infliximab was used at 5 mg/kg in weeks 0, 2, and 4.^444,445^ Compared to conventional treatment with methotrexate, infliximab has its advantages in treating plaque psoriasis.^446^ A prospective study showed that, compared with etanercept, infliximab exerted effects significantly faster before 24 weeks of treatment, but there were no significant differences in efficacy between the two medications at week 48.^447^ However, there are still some patients with psoriasis experiencing elevated liver enzymes and infusion reactions after using infliximab.^443–446^

Adalimumab, an IgG1 monoclonal antibody, has a similar mechanism of action to infliximab. Adalimumab can be used for different types of psoriasis and often has a prolonged effect. In a phase III study from REVEAL, 71% of the patients with plaque psoriasis achieved PASI 75 at week 16 after receiving treatment with adalimumab, while only 7% of the patients receiving placebo achieved PASI 75.^448^ Importantly, adalimumab can also be used in patients with poor response to etanercept (i.e., patients who did not reach PASI 50); according to a study, the PASI 75 response rates of such a patient group were 27%, 36%, and 54% at week 12, 24, and 48, respectively.^449^ Two other clinical trials showed an ACR 20 response rates of 65% and 52% after 12 weeks of treatment with adalimumab among patients with psoriatic arthritis.^450,451^ Of note, it was found that adalimumab was effective on nail psoriasis, with a NAPSI 75 response rate of 46.6% at week 26, compared to only 3.4% in the placebo group.^425^ According to multiple clinical trials, common adverse reactions of adalimumab include upper respiratory tract infection, rhinitis, and pain at the injection site.^425,448–451^

TNF-α inhibitors, the oldest biologics used to treat psoriasis, have been demonstrated to play an important role in different types of psoriasis. However, it is important to note that some patients with psoriasis developed worsening of psoriasis symptoms or new paradoxical psoriasis after the use of TNF-α inhibitors, using TNF-α inhibitors, which were called paradoxical reactions.^452^ This phenomenon appears to be associated with uncontrollable increased type 1 interferon cytokines, heightened number of Th-1 and Th17 cells, as well as individual genetic susceptibilities.^80,453^ If paradoxical reactions occur, TNF-α inhibitors will discontinue and change to biologics acting on other pathways, such as ustekinumab, an IL-12/23 antagonist.^452^

### IL-17 inhibitors

IL-17 is a key mediator in the development of psoriasis, and IL-17 inhibitors can treat psoriasis by binding to its ligands or receptors to block the IL-17 pathway.^30^ To date, three biologic agents targeting the IL-17 cytokine pathway have been approved to treat psoriasis by the FDA, and they include two monoclonal antibodies against interleukin-17A with high affinity, secukinumab and ixekizumab.

Secukinumab is the first monoclonal antibody among IL-17 inhibitors, and it was approved by the FDA in 2015 for the treatment of psoriasis. A randomized, double-blind clinical study showed that secukinumab could reverse the histopathology of plaque psoriasis by reducing IL-17A production, leading to plaque resolution.^454^ Two key clinical studies, ERASURE and FIXTURE, found that 77.1% and 81.6% of the patients given 300 mg of secukinumab and 67.0% and 71.6% of the patients given 150 mg of secukinumab achieved PASI 75 at week 12; notably, both doses of secukinumab showed better clinical effect than etanercept at a dose of 50 mg.^44^ In addition to being effective on plaque psoriasis, secukinumab has also proven to be effective in nail psoriasis and psoriatic arthritis. A study showed that 54.0% and 35.0% of the patients with psoriatic arthritis treated with 300 mg of secukinumab achieved ACR 20 and ACR 50 at week 12, respectively. Furthermore, secukinumab is found to have higher treatment retention than adalimumab in the treatment of psoriatic arthritis.^424^ Secukinumab is also effective in the treatment of nail psoriasis, which is demonstrated by a mean NAPSI improvement of −45.3% at a dose of 300 mg at week 16 and −63.2% at the same dose at week 32, respectively.^424^ With a similar mechanism to secukinumab, ixekizumab has similar efficacy in the treatment of psoriasis. A phase III trial showed that at least 87.0% of the patients with moderate-to-severe plaque psoriasis achieved a PASI 75 response after receiving continuous treatment of ixekizumab; it is also demonstrated that ixekizumab is superior to etanercept in clinical efficacy.^455^ However, secukinumab and ixekizumab are associated with adverse events such as infection, headache, neutropenia, and inflammatory bowel disease.^44,455,456^

In addition to two IL-17A inhibitors, brodalumab, an antibody directly against IL-17RA, has been used in treating psoriasis.^30^ Two phase III studies (AMAGINE-2 and AMAGINE-3) showed that, after the use of brodalumab via subcutaneous injection at 210 mg once every other week, the PASI 75 response rates were 85.0% and 86.0% at week 12 of treatment.^457^ In addition to plaque psoriasis, brodalumab is also effective in the treatment of articular psoriasis.^458,459^

Apart from the three types of IL-17 antibodies approved by the FDA, a new medication, bimekizumab, has already been approved in Europe as of August 2021 and in Japan as of January 2022, is waiting for approval.^460–462^ It is a monoclonal IgG1 antibody that specifically inhibits IL-17F and IL-17A.^30^ In a multicenter, double-blind, placebo-controlled, randomized withdrawal phase III trial, 91% of the patients with plaque psoriasis achieved PASI 90 response at 16 weeks after the start of bimekizumab administration at 320 mg once every 4 weeks and 89.8% of the patients achieved an Investigator Global Assessment (IGA) score of 0 or 1, indicating that the bimekizumab had faster and stronger effect compared the IL-17 and IL-23 inhibitors used in prior studies, making it a promising new IL-17 inhibitor.^460^ Bimekizumab is also associated with an excellent response rate in this study, with the relief of skin and joint symptoms lasting through 56 weeks.^46,460,463^ A recent study has revealed that blimekizumab and adalimumab have similar effects in the treatment of psoriatic arthritis and plaque psoriasis and that bimekizumab is associated with better skin clearance than secukinumab.^464^ However, blimekizumab is also associated with a higher risk of oral candidiasis. Thus, further studies are needed to weigh its therapeutic values against safety issues.^464,465^

### IL-12/IL-23p40 inhibitors

Ustekinumab was approved by the FDA for the treatment of psoriasis in 2009 and is currently the only IL-12/IL-23p40 inhibitor for this disease.^466^ It is a monoclonal antibody that specifically binds to the shared p40 subunit of IL-12 and IL-23, blocking the signaling pathways that are activated by normal ligand-receptor binding. In two phase III clinical trials, PHOENIX 1 and PHOENIX 2, the PASI 75 response rates were 66.7% and 67.1% at 12 weeks after the use of ustekinumab at 45 mg and 66.4% and 75.7% at 12 weeks after the use of ustekinumab at 90 mg (both doses were administered at week 0, week 4, and once every 12 weeks thereafter). Moreover, the maintenance of effect of ustekinumab is incredibly long, with a substantial response rate even in the third year after the start of treatment.^467,468^ Some studies showed that, after 12 weeks of standard treatment at a therapeutic dose for psoriasis, ustekinumab had a higher response rate than etanercept but a lower response rate than brodalumab.^47,457^ In addition to treating plaque psoriasis, ustekinumab is highly effective for psoriatic arthritis. According to a study, a dose of 90 mg of ustekinumab achieved an ACR 20 response rate of 49.5% after 24 weeks of treatment, compared to 22.8% of the placebo group.^469^ The strength and duration of the effect of ustekinumab in the treatment of psoriatic arthritis are similar to those of IL-17 inhibitors, such as secukinumab.^470,471^ Ustekinumab is also effective in treating psoriatic nail diseases, which is demonstrated by a significantly improved NAPSI after 24 weeks of treatment.^472^ Common adverse reactions to ustekinumab include upper respiratory tract infection, nasopharyngitis, neutropenia, and headache, and it has been found that ustekinumab has fewer adverse effects than TNFα inhibitors.^473^ At the same time, when paradoxical reactions occur in psoriasis treated with TNF-α inhibitors, switching to ustekinumab may be considered to treat psoriasis and the symptoms of paradoxical reactions that occur.^474^

### IL-23 inhibitors

Monoclonal antibodies that specifically inhibit IL-23 can directly reduce the production of psoriasis-related cytokines. At present, IL-23 antagonists mainly include guselkumab, tildrakizumab, risankizumab, and mirikizumab. Guselkumab is the first IL-23 inhibitor approved by the FDA in 2017 for the treatment of moderate to severe plaque psoriasis,^475^ and its clinical effect has been demonstrated in different types of psoriasis.^476–478^ VOYAGE 1/2 is the first phase III clinical trial to investigate guselkumab in the treatment of patients with psoriasis; in this study, patients were randomized to receiving placebo, adalimumab, or guselkumab at 100 mg in week 0, week 4, and once every 8 weeks thereafter. The results showed that 85% of the patients using guselkumab achieved an IGA score of 0 or 1 at week 16, and 73% achieved PASI 90 response, which was significantly superior to placebo and adalimumab; however, guselkumab did not show any significant difference in the incidence of adverse reactions from other treatment options.^45,479^ Tildrakizumab, a humanized monoclonal antibody that can significantly improve plaque psoriatic lesions, has demonstrated by stable efficacy and good tolerability. It also has less effect on risk factors for cardiometabolic diseases, indicating a better safety profile.^480–482^ Moreover, cost-effectiveness analyses of biologics have shown that tildrakizumab is one of the most cost-effective first-line treatment options for moderate to severe psoriasis.^483,484^ Risankizumab, a humanized IgG1 monoclonal antibody directly against the p19 subunit of IL-23,^485^ has also demonstrated a better therapeutic effect compared to ustekinumab and adalimumab.^486,487^ It seems that risankizumab has better efficacy and lower risk compared to other biologics acting on IL-23 p19, IL-12/IL-23 p40 and IL-17.^488^

### IL-36/IL-1 inhibitors

As shown above, the IL-36/IL-1 axis plays an important role in psoriasis, especially in GPP. To date, two humanized IgG monoclonal antibodies targeting the IL-36 receptor (IL-36R), spesolimab and imsidolimab, have demonstrated great efficacy in international clinical trials in patients with GPP.^110,489^ Spesolimab is a novel humanized selective antibody that blocks the IL-36R and the first medication under development to specifically target the IL-36 pathway for the treatment of acute GPP.^490^ In a phase I clinical trial on spesolimab, all 7 patients who received a single dose of spesolimab (10 mg/kg) intravenously showed rapid lesion improvement within 4 weeks.^491^ On this basis, the therapeutic value of spesolimab in GPP was further confirmed by the phase II Effisayil-1 study, which showed that 84.4% of the 35 patients with moderate to severe acute GPP had no visible pustules on the skin after 12 weeks of treatment and that 81.3% achieved clearance or complete clearance of skin symptoms.^492^ Imsidolimab also demonstrated significant efficacy in the phase II clinical trial GALLOP and has now entered phase III clinical trials.^110^ In addition to IL-36R, other parts of the IL-1/IL-36 axis are also the focus of studies. Anti-IL-1 agents such as anakinra, canakinumab, and gevokizumab have demonstrated good efficacy in psoriasis. Anakinra is a recombinant IL-1 receptor antagonist (IL-1Ra) that inhibits IL-1α and IL-β, and it has shown good effect in treating pustular psoriasis and deficiency in IL-1 receptor antagonist gene variants.^493,494^ Canakinumab is also an anti-IL-β antibody with good effects in GPP.^495^ Gevokizumab is another novel IL-1β antagonist, the effect of which has been preliminarily shown in a study, where two patients with GPP had 79% and 65% improvement in GPP score after 4 weeks of treatment.^496^ Therefore, IL-1 inhibitors may be effective in the treatment of psoriasis, especially GPP. But large-scale, prospective, randomized clinical trials are needed to confirm the efficacy and safety of these medications in the treatment of acute GPP. Moreover, it was found that IL-8 was significantly decreased in patients with refractory psoriatic arthritis treated with exogenous IL-36Ra, suggesting that the IL-36 axis is among the inflammatory pathways that are most relevant to refractory psoriatic arthritis.^497^ However, there is no clinical data showing the clinical efficacy of IL-36R-targeted therapies in psoriatic arthritis.

### JAK inhibitors

JAK inhibitors are a novel class of immunosuppressive agents that inhibit gene transcription of pro-inflammatory cytokines by blocking the JAK/STAT-mediated intracellular signaling pathways. Tofacitinib is a first-generation JAK inhibitor that mainly targets JAK3, JAK2, and JAK1 and blocks the JAK signal transduction pathway by binding to the ATP-binding site in the JAK protein, thereby relieving psoriatic arthritis. A Phase III study showed that 54% of the patients using tofacitinib at 5 mg twice daily and 63% of the patients using tofacitinib at 10 mg twice daily achieved ACR 20 response at month 3.^450^ Furthermore, it was approved for the treatment of psoriatic arthritis by FDA.^498,499^ Despite demonstrating superiority over placebo in treating moderate-to-severe plaque psoriasis, tofacitinib is not approved by the FDA for this indication due to concerns regarding its clinical efficacy and long-term safety.^500^ Upadacitinib, an oral JAK1 inhibitor approved in 2022 for the treatment of psoriatic arthritis, has also demonstrated good efficacy and safety profile in clinical trials.^451,501^ However, at present, no clinical trials have been designed to evaluate its efficacy and safety in psoriasis. In addition, baricitinib mainly acts on JAK1 and JAK3 and downregulates cytokines such as interferon-γ, IL-12, IL-17, and IL-23,^502,503^ and ruxolitinib acts on JAK1 and JAK2, thereby inhibiting the phosphorylation of STAT3 and promoting Th17 cell apoptosis^504^; both of them are first-generation JAK inhibitors and are effective for the treatment of psoriasis. However, all the first-generation JAK inhibitors inhibit the kinase portion of several JAK proteins, increasing the risk of adverse effects such as infection, hemoglobin reduction, thrombocytopenia, gastrointestinal perforation, and nausea.^505,506^ Second-generation JAK inhibitors can selectively inhibit individual JAK proteins without affecting other cytokines; for example, deucravacitinib, PF06826647, and brepocitinib selectively act on TYK2.^507–510^ Recently, a randomized, double-blind, placebo-controlled phase III clinical trial showed that the PASI 75 response rate reached 58.4% after 16 weeks of treatment with oral deucravacitinib at 6 mg daily, as compared to 12.7% of patients receiving placebo and 35.1% of patients receiving apremilast, with an incidence of adverse reactions similar to that of apremilast.^507^ Deucravacitinib was approved for the treatment of plaque psoriasis by the FDA in 2022. Although most selective JAK inhibitors are still under investigation and yet to be approved for marketing, they are regarded as promising medications due to their better safety profile.

### PDE4 inhibitors

Phosphodiesterase 4 (PDE4) can hydrolyze cyclic adenosine monophosphate (cAMP) and plays an important role in many biological processes. Owing to its broad anti-inflammatory activities, it has been investigated that PDE4 inhibitors can be applied for treating various skin disorders or rheumatic diseases, like psoriasis, PsA, and AD.^511^ Apremilast, an orally administered PDE4 inhibitor, was approved in the USA in 2014 for adult patients with active psoriatic arthritis or patients with moderate-to-severe plaque psoriasis who were candidates for phototherapy or systemic therapy. Since a PALACE 1 study investigated that a dose of 30 mg twice a day of apremilast achieved an ACR 20 response rate of 40.0% at week 16, compared to 19.0% of the placebo group,^512^ another phase III clinical trial revealed its efficacy in moderate to severe plaque psoriasis. It is shown that the PASI 75 response rates were 33.1% at week 16 after the use of apremilast at 30 mg twice a day, as compared to 5.3% of patients receiving placebo.^513^ However, the application of PDE4 inhibitors for the treatment of psoriasis has been hampered by their side effects, such as emesis. After great efforts, based on the DERMIS-1 and DERMIS-2 randomized clinical trials, in 2022, the FDA approved roflumilast as a cream to treat plaque psoriasis, including in skin folds, in patients aged 12 years and older.^514^ The clinical trials showed that 37.5–42.4% of the patients with plaque psoriasis after 8 weeks of treatment achieved Investigator Global Assessment (IGA) success (clear or almost clear status plus ≥ 2-grade improvement from baseline [score range, 0–4]). Further research is warranted to evaluate the comparative efficacy with other active treatments and to ascertain the long-term effectiveness and safety.

### RorγT antagonists

RORγt, mainly expressed in lymphocytes, can regulate the differentiation of CD4^+^ T cells into Th17 cells, which is closely related to psoriasis. Thus, RORγT inhibition might be an effective strategy for the treatment of psoriasis. VTP-43742 is an oral RORγT inhibitor; in a phase IIa study, a reduction by 29% and 23% in PASI was observed at 4 weeks of VTP-43742 use at 700 mg and 350 mg.^515^ Other oral RORγT inhibitors under investigation include JTE-451 and ABBV-157, and RORγT inhibitors are regarded as potential candidates for the treatment of psoriasis.^516^

### IL-22 inhibitors

IL-22 may be a new target for the treatment of psoriasis. However, a current clinical study (NCT01010542) on ILV-095, a medication targeting IL-22, was terminated prematurely because the primary efficacy endpoints could not be met. In this regard, we analyzed the reasons for the failure of clinical therapies targeting IL-22. This may indicate that IL-22 plays a protective effect against psoriasis. It has previously been reported that IL-22 can reduce nonalcoholic steatohepatitis by blocking hepatocyte oxidative stress.^517^ Since elevated ROS in keratinocytes can lead to keratinocyte proliferation and inflammation,^518^ Whether IL-22 may inhibit oxidative stress in keratinocytes are remaining to be clarified. Besides, it is reported that IL-22 helps maintain the homeostasis of the host immune system and induces immune tolerance by preventing colonization by pathogenic microorganisms.^519^ Considering the dysregulation of gut microbiota plays an important role in psoriasis,^520^ we hypothesize that the dysfunction of gut microbial homeostasis may be another reason for the failure of targeting IL-22.

### IFN inhibitors

Type I IFN may play a role in the pathogenesis of psoriasis;^521^ However, according to studies, an IFN-α agonist, MEDI-545, has demonstrated no treatment for plaque psoriasis. This may be related to MEDI-545, which targets IFN-α only. However, besides IFN-α, other types of Type I IFN, like IFN-β, can also participate in the development of psoriasis by promoting Th17 cell activation. Moreover, this could also reflect from the profile that IFN-α and even Type I IFN may not play a major role in psoriasis.

## Other clinical research progress

Mesenchymal stem cells (MSCs) are adult pluripotent progenitor cells with low immunogenicity, great self-renewal ability, and strong immunomodulatory properties.^522,523^ Thus, they are considered a treatment strategy for a variety of autoimmune diseases.^524,525^ It has been found that MSCs exhibited abnormal apoptosis, proliferation, differentiation, and cytokine secretion in patients with psoriasis.^526,527^ Furthermore, MSCs can activate the PI3K/AKT signaling pathway to stimulate the proliferation, differentiation, and migration of keratinocytes.^528^ All the above findings indicated the role of MSCs in the pathogenesis of psoriasis. By co-culturing MSCs of healthy donors with MSCs from patients with psoriasis, Campanatiet et al. found that inflammatory factors such as IL-17A and TNF-α were significantly reduced, which supported the therapeutic value of MSCs.^529^ In a phase I/IIa, single-arm study on human umbilical cord-derived MSCs (UMSCs), 47.1% of the 17 patients with psoriasis had at least 40% improvement in the PASI score during the treatment with UMSC infusion and through 6 months of follow-up, with a significant increase in Tregs and CD4^+^ memory T cells and a significant decrease in Th17 and CD4+ naive T cells in the peripheral blood after UMSC transplantation.^530^ The efficacy of MSCs in psoriasis may be superior to conventional treatments; however, because no large-scale clinical trials on this approach have been conducted, the type, mode, dose, and frequency of MSCs infusion are still under exploration.

The aryl hydrocarbon receptor (AhR) is a transcription factor expressed in skin cells such as keratinocytes, mast cells, and melanocytes.^531–533^ AhR signaling has been shown to regulate Th17 and Th22 cell differentiation, as well as the expression of IL-17 and IL-22,^534,535^ which have been found to increase in the psoriatic skin in AhR-deficient mice.^536^ Tapinarof is a topical aryl hydrocarbon receptor modulator separated from a variety of symbiotic bacterial metabolites. It is the first topical cream targeting AhR for psoriasis approved by the FDA.^537^ In a phase IIa, multicenter, open-label trial, tapinarof showed a certain effect in patients with plaque psoriasis. A study showed that, among 21 patients who received tapinarof cream 1% on a daily basis for 29 days, 18 patients had improved PASI scores, and 7 patients reached PASI 75 response.^538^ Later, two phase III multicenter, randomized, double-blind, vehicle-controlled trials were conducted on the efficacy and safety of tapinarof; in PSOARING 1, 35.4% of patients using tapinarof (vs. 6.0% of patients who received vehicle cream) daily for 12 weeks achieved the primary endpoint, and in PSOARING 2, the percentage was 40.2% (vs. 6.3% of patients who received vehicle cream).^539^ In addition, the incidence of adverse reactions, such as burning, stinging, and itching, was low in both trials. Benvitimod cream 1%, a preparation developed in China, has a similar mechanism of action to tapinarof cream 1%; it was approved in China in 2019 for topical use to treat mild-to-moderate plaque psoriasis and is usually used twice daily.^540,541^

S1P, acting on S1P receptor (S1PR) 1–5, has attracted widespread interest due to its inhibition effect on epidermal cell growth, induction of keratinocyte differentiation, and antiproliferative and anti-inflammatory effects in mouse models of psoriasis.^292,542^ Ponesimod, a selective S1PR1 agonist for oral use, demonstrated good efficacy in a phase II clinical trial, where 46.0% of patients on ponesimod at 20 mg once daily achieved PASI 75 response at week 16, 48.1% of patients on ponesimod at 40 mg once daily achieved this goal, and the percentage was only 13.4% in patients on placebo.^294^ The mechanism of S1PR agonist against psoriasis might be related to the reduction of white blood cell count, regional lymph node weight, IL-23 mRNA levels, and CD3^+^ T cell percentage.^543^ Known adverse reactions to ponesimod include dyspnea, elevation of liver enzymes, and dizziness; however, as ponesimod has not been intensely studied, its serious side effects still need to be investigated in further studies.^294^

While traditional therapy and biological agents targeting various inflammatory pathways have successfully treated psoriasis, certain patients still do not respond well. Various clinical studies are underway (Table 3). However, the efficacy is not sure due to the limited sample size and inadequate study duration of the trials. It is necessary to invest more in promising clinical trials. In Table 4, we discussed the advantages and limitations of all therapies available for psoriasis. And it is crucial to keep finding more effective ways to treat psoriasis. Recent studies have shown that metabolism plays an important role in psoriasis. It may be a promising method to focus on targeting psoriasis metabolism and specific metabolic mechanisms in the future. Moreover, an increasing number of research have shown potential connections between psoriasis and gut microbiota. Patients with psoriasis have reduced beneficial microbes in the gut, and gut microbes may cause skin disease by affecting immune balance. There is still insufficient research on the relationship between gut microbiota and psoriasis. Supplementing with probiotics and fecal transplantation also may be promising treatments for psoriasis. More clinical trials are expected to explore their efficiency in the treatment of psoriasis.

**Table 3:** Clinical trials of other psoriasis treatment

Target	Drug	Dose	Efficacy		NCT number
RORγT	JTE-451	200 mg twice daily	PASI 75	11.8% at week 16	03832738
		400 mg twice daily		22.0% at week 16	
	Mesenchymal stem cells	UMSC infusions once every 2 weeks	PASI 75	35.3% at month 6	03765957
			PASI 90	17.6% at month 6	
AhR	Topical tapinarof	1% twice daily	PASI 75	65.0% at week 12	02564042
		1% once daily		56.0% at week 12	
		0.5% twice daily		46.0% at week 12	
		0.5% once daily		46.0% at week 12	
		1% once daily	PASI 75	36.1% at week 12	03956355
			PASI 90	18.8% at week 12	
			PASI 75	43.6% at week 12	03983980
			PASI 90	20.9% at week 12	
S1PR1	Ponesimod	20 mg once daily	PASI 75	46.0% at week 16	01208090
			PASI 90	14.3% at week 16	
		40 mg once daily	PASI 75	48.1% at week 16	
			PASI 90	24.8% at week 16	

*AhR* Aryl hydrocarbon receptor, *S1PR1* S1P receptor 1, *UMSC* umbilical cord-derived mesenchymal stem cells, *AhR* aryl hydrocarbon receptor, *S1PR1* sphingosine 1-phosphate receptor 1

**Table 4:** Advantages and limitations of psoriasis therapy

Therapies		Advantages	Limitation
**Traditional therapy**			
Topical corticosteroids		Used for mild or localized psoriasis; Used for the acute phase of active psoriasis;	Long-term daily use of high-potency corticosteroids over large body surface areas is avoided in children. Rebound can occur from abrupt withdrawal. Common adverse reactions of topical corticosteroids include skin folliculitis, striae, telangiectasia, and atrophy.
Topical Vitamin D Analogues		Used liberally as long as the patient does not have renal impairment.	Modest efficacy when used lonely. Common adverse reactions include edema, burning, dryness, pruritus, erythema, and peeling. The rare adverse reaction includes hypercalcemia and parathyroid hormone suppression when exceeding the recommended dose.
Combination of topical corticosteroids and calcipotriol		Higher efficacy, fewer adverse effects, and longer remission than a monotherapy	Skin irritation occurs infrequently.
Topical calcineurin inhibitors		The important treatment methods for localized psoriasis. Used on thinner skin, such as facial and intertriginous areas; Used as steroid-sparing agents for prolonged use;	Common adverse reactions include burning and pruritus.
Topical keratolytic agents		One of the important treatment methods for localized psoriasis. Topical preparations with few systemic adverse reactions.	Common adverse reactions include erythema, burning, and pruritus. Tazarotene is avoided in pregnancy; Salicylic acid should exercise caution in children and patients with renal disease or hepatic disease; Salicylic acid should not be applied before phototherapy.
Coal tar		–	Rarely used due to its mutagenicity.
Methotrexate		The most commonly used treatment option before introducing biologics for moderate-to-severe psoriasis.	Adverse reactions include hepatotoxicity and severe gastrointestinal reactions, pneumonitis, pulmonary, fibrosis, bone marrow suppression, and teratogenicity. Contraindicated in case of renal insufficiency.
Cyclosporine		One of the important treatment methods for moderate-to-severe psoriasis. More effective than methotrexate in treating plaque psoriasis.	Adverse reactions include more severe renal toxicity, hypertension, hypomagnesemia, hyperkalemia, hyperlipidemia, lymphoma, and skin cancer risks; Not suitable for long-term use due to irreversible nephrotoxicity.
Acitretin		Efficacy in various types of psoriasis, particularly in erythrodermic psoriasis and pustular psoriasis.	Contraindicated in women of childbearing age due to teratogenicity, others include dry skin, dyslipidemia, and elevation of transaminases.
Psoralen and UV-A (PUVA)		Higher lesion clearance and longer maintenance in plaque psoriasis compared to NB-UVB.	The development of skin cancer with long-term use. Common adverse reactions include burning, gastro, photoaging, intestinal upset, and pruritus.
Narrow-band ultraviolet B (NB-UVB)		Efficacy in plaque psoriasis. Safer than PUVA.	Common adverse reactions include pruritus, erythema, blistering, photoaging.
**Targeted therapy**			
**Target**	**Molecule**	**Advantages**	**Limitation**
TNF-α	Etanercept Infliximab Adalimumab	Significant efficacy in various types of psoriasis than traditional treatments. TNF inhibitors are preferred in targeted therapy for psoriatic arthritis. Patients with a history of concomitant inflammatory bowel disease (IBD) might benefit from TNF-a inhibitors (Infliximab; Adalimumab).	Common adverse reactions include upper respiratory tract infection, nasopharyngitis, headache, and infusion-related reactions. Specific adverse reactions include paradoxical reactions, multiple sclerosis (rare), exacerbation or new onset of CHF, and Hepatotoxicity (especially with infliximab). Avoid use in patients with demyelinating disease, hepatitis B, latent tuberculosis, serious infection, and advanced congestive heart failure.
IL-17	Secukinumab Ixekizumab Brodalumab Bimekizumab	Significant efficacy in plaque psoriasis, psoriatic arthritis, and nail psoriasis. Adverse reactions and paradoxical reactions occur less frequently than TNF-α inhibitors.	Common adverse reactions include infection, headache, neutropenia, and injection site reaction. Specific adverse reactions include an increased risk of mucocutaneous *Candida* infection and inflammatory bowel disease (IBD), increased liver transaminases (Secukinumab), suicidal ideation, and completed suicides (Brodalumab). Avoid use in patients with IBD, history of allergic reaction to the therapeutic agents or vehicle, and serious infection.
IL12/IL23p40	Ustekinumab	Significant efficacy in plaque psoriasis, psoriatic arthritis, and nail psoriasis. When paradoxical reactions occur in psoriasis treated with TNF-α inhibitors, switching to Ustekinumab may be considered to treat psoriasis and the symptoms of paradoxical reactions that occur.	Common adverse reactions include upper respiratory tract infection, nasopharyngitis, neutropenia, and headache. Specific adverse reactions include hypersensitivity reactions (anaphylaxis and angioedema). Avoid use in patients with a history of allergic reactions to the therapeutic agents or vehicle and serious infections. Less effective than TNF-a inhibitors for psoriatic arthritis.
IL-23p19	Guselkumab Tildrakizumab Risankizumab	Significant efficacy in plaque psoriasis, psoriatic arthritis, and nail psoriasis.	Common adverse reactions include nasopharyngitis, upper respiratory tract infection, headache, arthralgia, and back pain. Specific adverse reactions include increased liver transaminase levels (rare). Avoid use in patients with febrile illness and a history of allergic reactions to the therapeutic agents or vehicle.
IL-36/IL-1	Spesolimab Imsidolimab	Significant efficacy in generalized pustular psoriasis (GPP). Convenient mode of administration: oral.	Adverse reactions include drug-induced liver injury, urinary tract infection, arthritis, drug reaction with eosinophilia, and systemic symptoms. Its’ efficacy and safety are still under investigation.
JAK	Tofacitinib Upadacitinib Deucravacitinib	Significant efficacy in psoriatic arthritis (tofacitinib and upadacitinib). Significant efficacy in plaque psoriasis (deucravacitinib). Convenient mode of administration: oral.	Adverse effects include infection, hemoglobin reduction, thrombocytopenia, gastrointestinal perforation, and nausea. Rare adverse reactions include lymphoma (deucravacitinib). Most selective JAK inhibitors are still under investigation and yet to be approved for marketing.
PDE4	Apremilast Roflumilast	Significant efficacy in psoriatic arthritis (Apremilast) and plaque psoriasis (Apremilast and Roflumilast). A topical cream with low rates of adverse effects (Roflumilast)	The application of Apremilast is hampered by its side effect, emesis. The roflumilast cream efficacy compared with other active treatments and longer-term efficacy and safety need to be further assessed.
RORγt	VTP-43742	Potential candidates for the treatment of psoriasis.	Absence of adequate clinical study data to demonstrate efficacy and identify adverse reactions.
IL-22	ILV-095	A new target for the treatment of psoriasis.	Primary efficacy endpoints could not be met.
IFN-α	MEDI-545	–	Its’ efficacy still remains unclear.
**Other clinical research progress**			
–	Mesenchymal stem cells (MSCs)	Improvement of the symptoms of patients with psoriasis.	The absence of adequate clinical study data on the approach has been conducted, and the type, mode, dose, and frequency of MSCs infusion are still under exploration.
AhR	Tapinarof	Topical preparations with few systemic adverse reactions.	Low rate of adverse reactions, such as burning, stinging, and itching. Absence of adequate clinical study data to demonstrate efficacy and identify adverse reactions.
S1PR1	Ponesimod	A new target for the treatment of psoriasis. Convenient mode of administration: oral	Adverse reactions include dyspnea, elevation of liver enzymes, and dizziness. Absence of adequate clinical study data to demonstrate efficacy and identify adverse reactions.

## The recurrence of psoriasis

The aforementioned biologics have brought great benefits and new hope to patients with psoriasis. However, practical data suggest that psoriatic lesions often recur at the same sites after the discontinuation of the biologics,^544^ making disease recurrence one of the greatest challenges in the treatment of psoriasis.

**Fig. 7:**
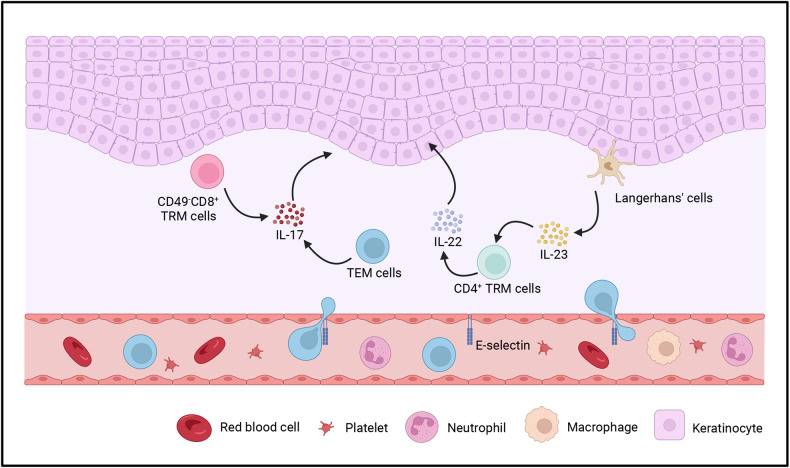
The current mechanistic model of recurrent psoriasis. In clinically resolved lesions, CD4^+^ TRMs remain in the dermis, while CD8^+^ TRMs and eLCs remain in the epidermis. With a disease trigger, eLCs release IL-23, leading to the release of IL-22 by CD4^+^ TRMs, thereby further inducing the hyperproliferation and inflammation of keratinocytes. CD49^–^ CD8^+^ TRM cells also produce IL-17 to drive recurrent local inflammation. After interacting with the vascular addressin E-selectin, TEM cells migrate to the skin and release IL-17, thereby participating in disease recurrence. Created with BioRender.com

A variety of factors, including skin resident memory T (TRM) cells, memory-like γδT cells, and keratinocytes with inflammatory memory, are all considered possible mechanisms for the recurrence of psoriasis (Fig. 7). Among them, TRM cells are recognized as the major driver of psoriasis relapse.^545^ It is reported that effectors including CD69, integrins (CD49a, VLA-1, CD103, αvβ6, and αvβ8), aryl hydrocarbon receptor (AhR), CCR7, and the CCL27-CCR10 axis are related with the retention of TRM cells.^546,547^ A study using high-throughput screening on the T cell receptor (TCR) and immunostaining found that IL-17-producing resident αβT cells with psoriasis-specific antigen receptors were increased in psoriatic lesions and resolved psoriatic lesions, but not in the skin of healthy individuals. These findings indicate that TRM cells exist in the skin even when the disease is resolved, and they can lead to disease recurrence once activated.^548^ TRM cells can be divided into CD8^+^ and CD4^+^ subsets. The majority of CD8^+^ TRM cells express the cell marker CD103 (also known as integrin alpha‐E), which can bind to the E‐cadherin of keratinocytes.^549^ With the CD8^+^ TRM cells further classified as CD49^+^CD8^+^ TRM cells and CD49^–^CD8^+^ TRM cells, studies found that it was CD49^–^CD8^+^ TRM cells that drove recurrent local inflammation by producing IL-17 in the presence of certain triggers.^550^ Skin‐resident CD4^+^ T cells can also drive keratinocyte pathology through IL‐22 production.^551^ In summary, CD8+ TRM cells (which produce IL17) together with CD4^+^ TRM cells (which secrete IL-22 after the stimulation of IL-23 produced by epidermal Langerhans cells) induce keratinocyte hyperproliferation and subsequent disease relapse in resolved lesions. In addition, it is reported that through the interaction with the vascular addressin E-selectin, effector memory T (TEM) cells migrate to the skin and release IL-17, thereby participating in disease relapse.^546^ Lastly, it is worth mentioning that recent studies have revealed that skin epithelial stem cells (EpSCs) or basal keratinocytes with stemness can acquire long-term epigenetic memory during the course of psoriasis, which may play a potential role in the recurrence of skin inflammation in psoriasis.^552^ However, the above potential mechanisms still need to be verified in future works.

## Conclusion and future prospects

Psoriasis is a common, chronic, inflammatory skin disease with a high burden on individuals, health systems, and society worldwide, suggesting the urgent need to comprehensively understand the mechanisms driving the pathogenesis of psoriasis. This review focused on altered signaling pathways involved in psoriasis pathogenesis. In the past decade, the immunopathology and pathogenesis of psoriasis have been gradually uncovered, and the development of therapeutic approaches has gained revolutionary progress. Targeted therapies with biologics have brought new hope to patients with psoriasis due to their effectiveness in modifying the disease or, potentially, even curing it by preventing the accumulation of tissue-resident memory T cells in the skin. Nevertheless, there are still many unsolved issues in psoriasis, with the adverse effects of medications and disease relapse after the discontinuation of treatment being the most prominent ones. With a deeper understanding of the mechanisms of disease recurrence, it is reasonable to believe that early treatment to prevent the emergence and spreading of issue-resident memory T cells is highly desirable. Latest research has found that IL-23 produced by CD301b^+^ cells can promote in situ proliferation of TRM cells, indicating that IL-23 inhibitors or new therapies targeting CD301b may be a promising strategy to cope with disease recurrence and to maintain the therapeutic effect.^553^ However, the clinical translation of novel therapeutic approaches still needs great efforts in the future.

Although great progress has been made in the understanding and treatment of psoriasis, further research is still needed. Future, whether genetic indicators and biomarkers can be used as predictors for psoriasis to facilitate early diagnosis and intervention is another major focus of future studies. Moreover, despite the discovery of multiple potential metabolic molecules, there is no comprehensive understanding of metabolic effects in psoriasis. With new multi-omics tools such as single-cell metabolomics and spatial metabolomics emergence, it is possible and necessary to construct a multi-dimensional metabolic network of psoriasis through algorithm innovation and multi-omics joint analysis. Targeting metabolism therapy is still limited, possibly related to metabolic heterogeneity, flexibility, and unpredictable adverse effects. Hence, identifying more desirable metabolic targets will be a crucial step forward. We believe that focusing on psoriatic metabolism and establishing a psoriasis network centered on metabolism will be the hot topic in the next decade. Another challenge in the future is to find effective therapies to manage psoriatic comorbidities, especially metabolic and cardiovascular disorders. Last but not least, attention is also needed to the relatively rare subgroups of psoriasis, such as palmoplantar psoriasis and pustular psoriasis, which are poorly responsive to current treatment options.
