# Correction: Signaling pathways and targeted therapies for psoriasis

**DOI:** 10.1038/s41392-023-01733-9

**Published:** 2024-01-22

**Authors:** Jia Guo, Hanyi Zhang, Wenrui Lin, Lixia Lu, Juan Su, Xiang Chen

**Affiliations:** 1 Department of Dermatology, Xiangya Hospital, Central South University, No. 87 Xiangya Road, Changsha, 410008 Hunan China; 2 National Engineering Research Center of Personalized Diagnostic and Therapeutic Technology, Changsha, 410008 Hunan China; 3 Hunan Key Laboratory of Skin Cancer and Psoriasis, Changsha, 410008 Hunan China; 4 Hunan Engineering Research Center of Skin Health and Disease, Changsha, 410008 Hunan China

**Keywords:** Inflammation, Immunopathogenesis, Therapeutics

Correction to: *Signal Transduction and Targeted Therapy* 10.1038/s41392-023-01655-6, published online 27 November 2023

After online publication of the article, the authors noticed an inadvertent error occurred in the third author’s given name. Also, the email of corresponding author is incorrect. The correct text is provided as follows:

1. ‘Wenri Lin’ should be corrected into ‘Wenrui Lin’
2. The email of Juan Su should be corrected as ‘sujuanderm@csu.edu.cn’

The original article has been corrected.

